# Changes in mortality due to Chronic Liver Diseases (CLD) during the COVID-19 pandemic: Data from the United States’ National Vital Statistics System

**DOI:** 10.1371/journal.pone.0289202

**Published:** 2024-09-03

**Authors:** James M. Paik, Dipam Shah, Katherine Eberly, Pegah Golabi, Linda Henry, Zobair M. Younossi

**Affiliations:** 1 Betty and Guy Beatty Center for Integrated Research, Inova Health System, Falls Church, VA, United States of America; 2 Center for Liver Disease, Department of Medicine, Inova Fairfax Medical Campus, Falls Church, VA, United States of America; 3 Center for Outcomes Research, Washington DC, United States of America; 4 Inova Medicine, Inova Health System, Falls Church, VA, United States of America; Samsung Medical Center, REPUBLIC OF KOREA

## Abstract

**Introduction:**

We assessed chronic liver disease (CLD)-related mortality in the U.S. using death data (2011–2021) obtained from National Vital Statistics System (NVSS). The average annual percentage change (AAPC) from the models selected by Joinpoint regression analysis over the pre-pandemic (2011–2019) and the 2019–2021 were reported because non-linear trend in death rates were observed over the 2011–2021. Liver-specific death was defined as an underlying cause of death and Chronic liver disease (CLD)-related death was defined as any cause of death. During the pre-pandemic, age-standardized HCC- and cirrhosis-specific death rates were annually increased by AAPC = +1.18% (95% confidence interval, 0.34% to 2.03%) and AAPC = +1.95% (1.56% to 2.35%). In contrast, during the 2019–2021, the AAPC in age-standardized cirrhosis-specific death rate (per 100,000) accelerated by up to AAPC +11.25% (15.23 in 2019 to 18.86 in 2021) whereas that in age-standardized HCC-specific death rate slowed to -0.39 (-1.32% to 0.54%) (3.86 in 2019 to 3.84 in 2021). Compared to HCC-specific deaths, cirrhosis-specific deaths were more likely to be non-Hispanic white (72.4% vs. 62.0%) and non-Hispanic American Indian and Alaska native (AIAN) (2.2% vs. 1.1%) and have NAFLD (45.3% vs. 12.5%) and ALD (27.6% vs. 22.0%). During the 2019–2021, the age-standardized HCV- and HBV-related death rate stabilized, whereas the age-standardized NAFLD- and ALD-related deaths rate increased to 20.16 in 2021 (AAPC = +12.13% [7.76% to 16.68%]) and to 14.95 in 2021 (AAPC = +18.30% [13.76% to 23.03%]), which were in contrast to much smaller incremental increases during the pre-pandemic (AAPC = +1.82% [1.29% to 2.35%] and AAPC = +4.54% [3.97% to 5.11%]), respectively). The most pronounced rise in the age-standardized NAFLD-related death rates during the pandemic was observed among AIAN (AAPC = +25.38%), followed by non-Hispanic White female (AAPC = +14.28%), whereas the age-standardized ALD-related death rates during the pandemic were highest among AIAN (AAPC = +40.65%), followed by non-Hispanic Black female (AAPC = +26.79%).

**Conclusions:**

COVID-19 pandemic had a major negative impact on cirrhosis-specific and CLD-related mortality in the U.S. with significant racial and gender disparities.

## Introduction

In March 2020, the World Health Organization (WHO) declared that Coronavirus-2019 (COVID-19) caused by the infectious agent, SARS-COv-2, a world-wide pandemic [1]. Over the past 3 years, COVID-19 was not only responsible for substantial direct global mortality and morbidity, but has also led to increases in deaths due to other chronic diseases to include chronic liver disease (CLD) [1].

In the United States (USA), the most common causes of CLD are viral hepatitis B and C (HBV and HCV), alcohol related liver disease (ALD), and nonalcoholic fatty liver disease (NAFLD). The U.S. Centers for Disease Control (CDC) estimates that 4.5 million adults have CLD (1.8% of the adult population) which is the 11^th^ leading cause of death [2, 3]. However, the etiology pattern of CLD is changing. Alcohol related liver disease (ALD) and non-alcoholic fatty liver disease (NAFLD) have become the predominant causes of CLD displacing viral hepatitis (hepatitis B virus and hepatitis C virus) [3–6]. The increasing burden of NAFLD is driven by the epidemics of obesity and type 2 diabetes (T2D) while curative drugs for HCV and vaccination as well as viral suppression drugs for HBV have led to a decrease in viral hepatitis. Reasons for the increase in ADL are multifaceted which vary by geographical area [3–6].

However, despite the few studies completed which have reported on the outcomes of patients with CLD who were hospitalized with COVID-19 [7–16], data assessing the impact of COVID-19 infection on CLD using a population-based data in the U.S is not available. These data could be important for future national and global strategies to deal with similar health emergencies. Therefore, we aimed to evaluate and quantify CLD-related mortality rates and cirrhosis-specific and hepatocellular carcinoma (HCC)-specific mortality rates in the U.S. with a focus on the trends before and during the COVID-19 pandemic using data from our National Vital Statistics System (NVSS) years 2011–2021.

## Methods

### Data sources

NVSS is part of the National Center for Health Statistics (NCHS) under the direction of the Centers for Disease Control (CDC). The data (2011–2021) were abstracted from public-use-multiple cause of death files [17, 18]. The NCHS annually compiles information from death certificates filed in all 50 states and D.C. into the NVSS system, in which cause of death is coded according to the *International Classification of Disease* (*ICD*) revision in use at the time of death (*ICD-8* for 1969–1978, *ICD-9* for 1979–1998, and *ICD-10* for 1999–2020). The underlying cause-of-death is defined by the World Health Organization as "the disease or injury which initiated the train of events leading directly to death, or the circumstances of the accident or violence which produced the fatal injury" [19]. Herein, multiple conditions not listed as underlying cause-of death were defined as contributory cause-of-death. Additionally, since NAFLD is typically under-coded in clinical practice (K76.0, K75.81), we used the published algorithm for identification of NAFLD to minimize its underestimation in our reported results [20].

To minimize ascertainment bias, using the published coding algorithm [18, 20], liver-specific deaths were identified as either 1) mortality caused by liver diagnosis as the underlying cause of death or 2) mortality caused by liver disease-related complications (eg. ascites, encephalopathy, hepatorenal syndrome, liver failure, sepsis, etc) as the underlying cause of death with liver diagnosis as the contributory cause of death. Of these, decedents with HCC as an underlying cause of death were defined as “HCC-specific deaths”; decedents with cirrhosis as the underlying cause of death were defined as “cirrhosis-specific deaths”; and all remaining decedents were classified as “other liver-specific deaths” which encompassed residual causes, including acute liver failure, cholangitis, and post-transplant liver failure [19, 20].

For CLD-related deaths, the following codes were used for each CLD that was investigated as an underlying or contributory cause of death: HBV codes B16, B18.0–1, B19.1; HCV codes B17.1, B18.2, B19.2, and K70 was mapped to ALD. As noted above, because NAFLD is typically under-coded in the clinical practice (K76.0, K75.81), we presumed that NAFLD also included those individuals who were coded for cryptogenic liver disease (K76.9, K74.6) in the absence of any codes for other causes of CLD such as HCV, HBV, ALD, autoimmune hepatitis, other inflammatory liver diseases, etc) or excessive alcohol use [5, 20, 21]. For CLD-related deaths, cause-specific death was also reported. **S1 Table in S1 File** displays the ICD-10 codes used. In contrast, comorbidities were defined as the corresponding ICD 10-codes in any cause of death via Elixhauser comorbidity index [22].

Socio-demographic variables included age at the time of death, sex, race, marital status, and college degree. We present combined single-race and Hispanic-origin categories consistent with the 1997 Office of Management and Budget standards [23] as non-Hispanic white, non-Hispanic black, non-Hispanic Hispanic American Indian and Alaska native (AIAN), non-Hispanic Asian including Pacific Islander, and Hispanic. Decedents for whom two or more races are included in totals but are not distributed among race and Hispanic-origin categories since the small number of multiple-race decedents were reported (<0.5% of deaths) in NVSS.

### Statistical methods

Age-standardized death rate were calculated by using the direct method, based on the census 2000 standard population by 10-year age groups [24, 25]. Joinpoint regression (JR) analysis was performed to assess temporal trends in age-standardized death rates using Joinpoint Regression Program version 5.0.1 (Statistical Research and Applications Branch, National Cancer Institute) because non-linear trend was observed over the entire study period. The JR model has several different lines connected at the joinpoints [26, 27]. The software enables to compare JR models by starting with no joinpoints (a straight-line trend) and subsequently testing whether 1 or more joinpoints needed to be entered into the JR model to best fit the data. Our analysis used a maximum number of 3 joinpoints, and the modified Bayes information criterion for model selection. We selected the most concise JR model to report the estimated annual percentage change (APC) for each detected time segment and the average annual percent change (AAPC) for the entire study period (2011–2021). The AAPC is a weighted average of APCs with weights equal to the length of the detected time segments. Since JP analysis detected a precipitous rise in death rate after 2019, we also reported AAPC over the pre-pandemic (2011–2019) and the 2019–2021 [26, 27].

The increasing or decreasing trend was defined if the AAPC was significantly different from 0; otherwise, a stable or level trend was defined. Subgroup analyses were conducted by sex and race/ethnicity. All analyses were performed with SAS software, version 9.4 (SAS Institute, Cary, NC). All research was conducted in accordance with both the Declarations of Helsinki and Istanbul, all research was approved by the appropriate ethics and/or Institutional review committee at Inova Health System, Falls Church, VA and was given a waiver of consent.

## Results

The number of deaths in the U.S. in 2020 and 2021 was 3,348,877 and 3,428,561, an increase of 18.7% and 21.6%, respectively as compared to deaths in 2019 (2,820,654) **(S2 Table in S1 File)**. Most of these increases (66.5% and 68.6%) could be attributed to COVID-19 which was noted as the underlying cause of death for a total of 351,307 and 417,252 deaths making it the 3rd leading cause of death in 2020 and 2021. Additionally, there were 103,795 liver-specific deaths in 2020 and 110,534 in 2021. This represented an increase of +8.8% and +15.9% from 95,380 liver-specific deaths in 2019 and making liver-specific deaths as the 8th leading cause of death in 2020 and 2021 according to NVSS data.

### Characteristics of liver-specific deaths

Cause-specific deaths were defined as an underlying cause of death. The characteristics of liver-specific deaths by HCC-, cirrhosis-, and other liver-specific deaths during 2011–2021 are provided in **S3 Table in S1 File**. Of a total of 1,012,372 liver-specific deaths (mean [SD] age, 63.7 [13.1] years; 62.9% male; 70.2% white; 10.4% black; 13.9% Hispanic, 3.2% Asian; and 1.9% AIAN), HCC- and cirrhosis-specific causes accounted for 11.6% and 42.7%, respectively. Compared to cirrhosis-specific deaths, HCC-specific deaths were more likely to be older (mean age: 67.2 vs. 62.0 years); male (77.6% vs. 62.5%); non-Hispanic Black (15.4% vs. 8.3%); non-Hispanic Asian (7.0% vs. 1.7%) and have HCV (22.3% vs. 4.4%) and HBV (2.8% vs. 0.3%). In contrast, cirrhosis-specific deaths were more likely to be non-Hispanic white (72.4% vs. 62.0%) and AIAN (2.2% vs. 1.1%) and have NAFLD (45.3% vs. 12.5%) and ALD (27.6% vs. 22.0%).

### Trend in age standardized liver-specific, HCC-specific and cirrhosis -specific death rates overall, by sex and by ethnicity from 2011 to 2021

**Fig 1:**
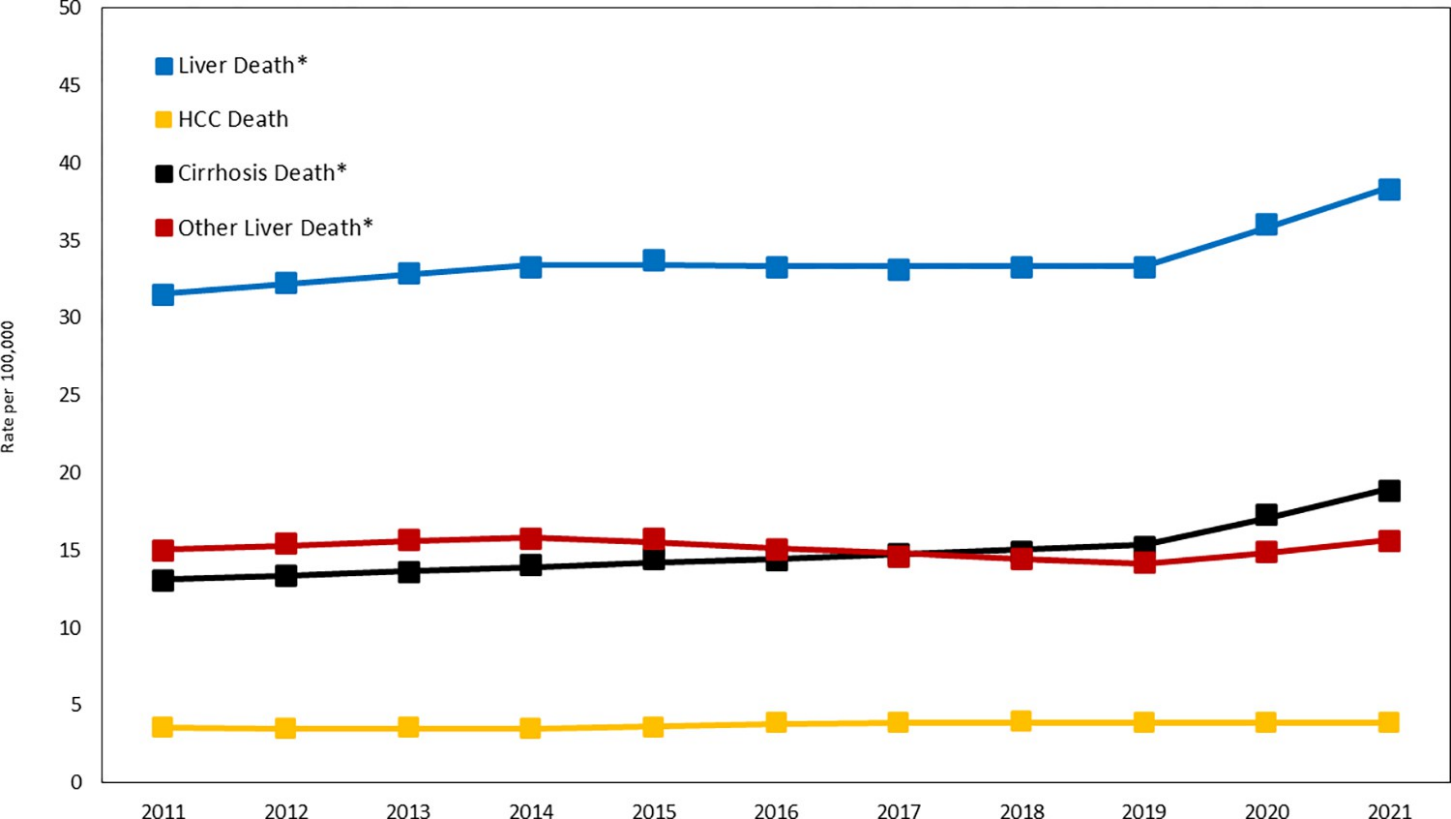
Trends in age-standardized liver-, Hepatocellular Carcinoma (HCC)-, and cirrhosis-specific death rate: United States, 2011–2021. * Significant increasing during the pandemic (2019–2021); p <0.05. NOTES: Data markers represent observed rates; lines are fitted rates based on joinpoint analysis. SOURCE: National Center for Health Statistics, National Vital Statistics System, Mortality.

**Overall trends.** Assessing liver-specific mortality in the past 10 years will give an opportunity to see changes in liver-specific mortality during the pandemic. In this context, the age-standardized liver-specific death rate (per 100,000) increased from 31.51 in 2011 to 33.30 in 2019 with an AAPC of +0.66% (95% confidence interval: 0.25% to 1.08%) and then sped up to 38.31 in 2021 with an AAPC of +7.46% (5.10% to 9.86%) **(Table 1 and Fig 1)**.

**Table 1:** Trends in age-standardized liver-specific death rate per 100,000 population by sex in the Unites States, 2011–2021.

		Counts (Rate per 100,000)			2011–2021	2011–2019	2019–2021
Sex	Cause of Death	2011	2019	2021	AAPC (95% CI)	AAPC (95% CI)	AAPC (95% CI)
Both	Liver Death	78,468 (31.51)	95,380 (33.3)	110,534 (38.31)	1.99 (1.55–2.42)	0.66 (0.25–1.08)	7.46 (5.1–9.86)
	HCC	8,997 (3.53)	11,872 (3.86)	12,122 (3.84)	0.87 (0.17–1.57)	1.18 (0.34–2.03)	-0.39 (-1.32–0.54)
	Cirrhosis	32,286 (13.02)	42,795 (15.24)	53,309 (18.86)	3.75 (3.18–4.32)	1.95 (1.56–2.35)	11.25 (7.89–14.71)
	Other liver	37,185 (14.96)	40,713 (14.2)	45,103 (15.61)	0.38 (-0.47–1.24)	-0.81 (-1.6 - -0.02)	5.27 (0.57–10.2)
Female	Liver Death	28,220 (21.31)	36,037 (23.75)	42,499 (28.12)	2.87 (2.6–3.13)	1.34 (1.08–1.6)	9.19 (7.81–10.59)
	HCC	1,958 (1.43)	2,706 (1.64)	2,785 (1.67)	1.96 (1.32–2.6)	1.96 (1.32–2.6)	1.96 (1.32–2.6)
	Cirrhosis	11,948 (9.1)	16,139 (10.87)	20,245 (13.74)	4.26 (3.43–5.11)	2.39 (1.8–2.98)	12.11 (7.2–17.24)
	Other liver	14,314 (10.78)	17,192 (11.24)	19,469 (12.72)	1.71 (0.84–2.58)	0.46 (-0.35–1.29)	6.82 (2.12–11.74)
Male	Liver Death	50,248 (42.99)	59,343 (44.1)	68,035 (49.53)	1.45 (0.79–2.12)	0.33 (-0.32–1.00)	6.04 (2.7–9.48)
	HCC	7,039 (5.96)	9,166 (6.44)	9,337 (6.35)	0.62 (0.03–1.21)	1.00 (0.29–1.72)	-0.91 (-1.69 - -0.12)
	Cirrhosis	20,338 (17.39)	26,656 (20.08)	33,064 (24.37)	3.37 (2.71–4.04)	1.71 (1.25–2.17)	10.33 (6.46–14.33)
	Other liver	22,871 (19.65)	23,521 (17.57)	25,634 (18.81)	-0.45 (-1.7–0.82)	-1.42 (-2.62 - -0.2)	3.53 (-2.95–10.44)

Abbreviation: AAPC, average annual percent change; HCC, Hepatocellular carcinoma; CI, confidence interval.

Standardized to the 2000 US standard population by 10-year age group.

AAPC was a weighted average of annual percentage change with weights equal to the length of the detected time segments from the model selected by Joinpoint regression analysis.

Liver-Specific Death was defined as an underlying cause of death

Before the pandemic (2011–2019), age-standardized HCC- and Cirrhosis-specific deaths were increasing with an AAPC of +1.18% (0.34% to 2.03%) from 3.53 in 2011 to 3.86 in 2019 and +1.95% (1.56% to 2.35%) from 13.02 in 2011 to 15.24 in 2019, respectively. During the 2019–2021, the AAPC in age-standardized cirrhosis-specific death rate (per 100,000) accelerated by up to +11.25% (7.89% to 14.71%) (18.86 in 2021) whereas that in age-standardized HCC-specific death rate slowed to -0.39 (-1.32% to 0.54%) (3.84 in 2021).

#### Sex

Overall trends in liver-, HCC-, and cirrhosis-specific death rates occurred similarly in males and females. Liver-specific death rates was consistently higher in males than in females. Notably, the increases in liver-specific death rates from 2019 to 2021 were faster in female [AAPC: +9.19 (7.81–10.59)] than in male [AAPC: +6.04, (2.7–9.48)] **(Table 1)**.

#### Ethnicity

From 2019 to 2021, the fastest annual increase in the age-standardized liver-specific death occurred in AIAN females (AAPC = +28.60% [16.88% to 40.27%]) and AIAN males (AAPC = +28.43% [12.14% to 47.09%], followed by white females (AAPC = +10.06% [7.86% to 12.30%]), white males (AAPC = +7.24% [3.06% to 11.59%]), Black females (AAPC = +8.48% [0.61% to 16.96%]), and Hispanic males (AAPC = 4.02% [1.72% to 6.38%]) (**S4 Table in S1 File)**. During the same period, observed changes in age-standardized liver-specific mortality for Hispanic female, Black male, and Asian were not significant. Similar patterns were observed for age-standardized cirrhosis-specific deaths. Of the note, excluding AIAN, Black females had the fastest annual increase in age-standardized cirrhosis-specific deaths (AAPC = +15.2% [3.78% to 27.80%]), twice as fast as black males (AAPC = +7.56% [0.45% to 15.18%]) **(S5 Table in S1 File)**. A finding most likely driven by differences between black females and black males in ALD-related deaths (AAPC = +26.79% [9.72% to 46.51%] black females vs. AAPC = +19.4% [11.60% to 27.74%] for black males) and NAFLD-related deaths (AAPC = +10.76% [4.30% to 17.62%] for black females vs. AAPC = +7.73% [2.62% to 13.10%] for black males). In contrast, during the 2019–2021, age-standardized HCC-specific death stabilized in most races but increased slightly in AIAN (AAPC = +1.89% [0.10% to 3.71%]) and decreased in Asian (AAPC = -2.74% [-4.56% to -0.89%]) **(S6 Table in S1 File)**.

### Summary liver-specific death rate results for 2021 by sex and ethnicity

In 2021, the male-to-female ratios for liver-, HCC-, and cirrhosis-specific death rates in 2021 were 1.56, 3.86, and 1.46, respectively. By ethnicity the liver-, HCC-, and cirrhosis-specific death rates per 100,000 were highest for AIAN (152.06, 6.75, and 85.98), followed by Hispanic (46.5, 5.57 and 23.73), white (38.72, 3.32, and 19.65), black (33.01, 4.95. and 13.19), and Asian (22.50, 5.08, and 5.89).

### Characteristics of CLD-related deaths

CLD-related deaths were defined as either an underlying or contributory cause of death. The characteristics of CLD-related deaths are available in **S7 Table in S1 File**. During the 2011–2021, there are 479,011 decedents with NAFLD, 290,952 ALD, 189,398 HCV and 19,270 HBV. Compared to ALD-, HCV-, and HBV-related deaths, NAFLD-related deaths were more likely to be older (65.7 vs. 56.9, 60.7, and 61.5 years, respectively), females (41.7% vs. 29.6%, 28.3%, and 27.1%), non-Hispanic white (73.2% vs. 71.0%, 63.1%, and 45.2%), and to have cirrhosis (78.8% vs. 77.9%, 56.0%, and 49.2%), congestive heart failure (11.1% vs. 5.2%, 6.9%, and 6.1%), cardiac arrhythmias (5.3% vs. 3.0%, 3.9%, and 4.3%), and renal failure (11.6% vs. 6.9%, 9.0%, and 9.7%). Compared to HCV- and HBV-related deaths, ALD-related deaths were more likely to be younger, Hispanic (15.8% vs. 14.1%, and 7.8%) and AIAN (3.7% vs. 1.7% and 0.8%) and to have alcohol abuse (89.4% vs. 19.8% and 11.9%). Compared to NAFLD- and ALD-related deaths, HCV- and HBV-related deaths were more likely to be non-Hispanic Black (18.2% and 18.6% vs. 8.7% and 7.6%) and HCC (16.3% and 19.0% vs. 3.9% and 2.2%). Compared to decedents NAFLD-, ALD-, and HCV-related deaths, HBV-related deaths were more likely to be Asian (27.0% vs. 2.2%, 1.4%, and 2.3%).

### Patterns of CLD-related death from 2011 to 2021

**Fig 2:**
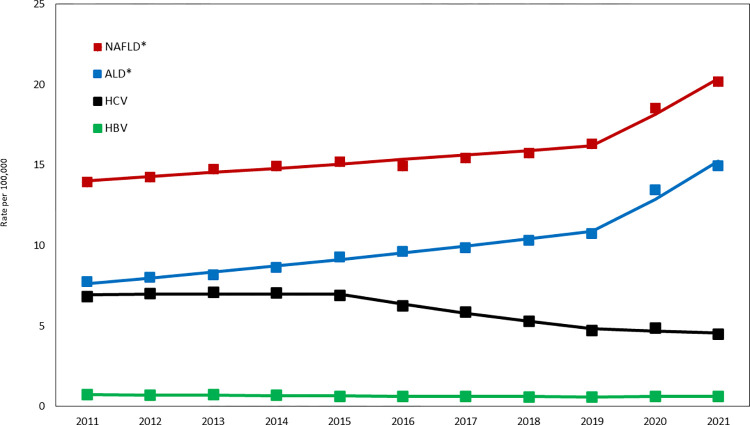
Trends in age-standardized CLD-related death rate: United States, 2011–2021. * Significant increasing during the pandemic (2019–2021); p <0.05. NOTES: Data markers represent observed rates; lines are fitted rates based on joinpoint analysis. SOURCE: National Center for Health Statistics, National Vital Statistics System, Mortality.

Before the pandemic (2011–2019), the age-standardized NAFLD- and ALD-related death rate were increasing from 13.93 in 2011 to 16.32 in 2019 (AAPC = +1.82% [1.29% to 2.35%]) and from 7.76 in 2011 to 10.74 in 2019 (AAPC = +4.54% [3.97% to 5.11%]), respectively **(Fig 2 and S8 and S9 Tables in S1 File)**. In contrast, the age-standardized HCV- and HBV-related death rates were decreasing from 6.83 in 2011 to 4.72 in 2019 (AAPC = -4.48% [-5.66% to -3.29%]) and from 0.72 in 2011 to 0.59 in 2019 (AAPC = -2.68% [-3.36% to -0.07%]), respectively **(Fig 2 and S10 and S11 Tables in S1 File)**. From 2019 to 2021, the age-standardized HCV- and HBV-related death rate leveled, whereas the age-standardized NAFLD- and ALD-related deaths rate soared to 20.16 in 2021 (AAPC = +12.13% [7.76% to 16.68%]) and to 14.95 in 2021 (AAPC = +18.30% [13.76% to 23.03%]), respectively, which were in stark contrast to the incremental increase between 2011 and 2019.

**Fig 3:**
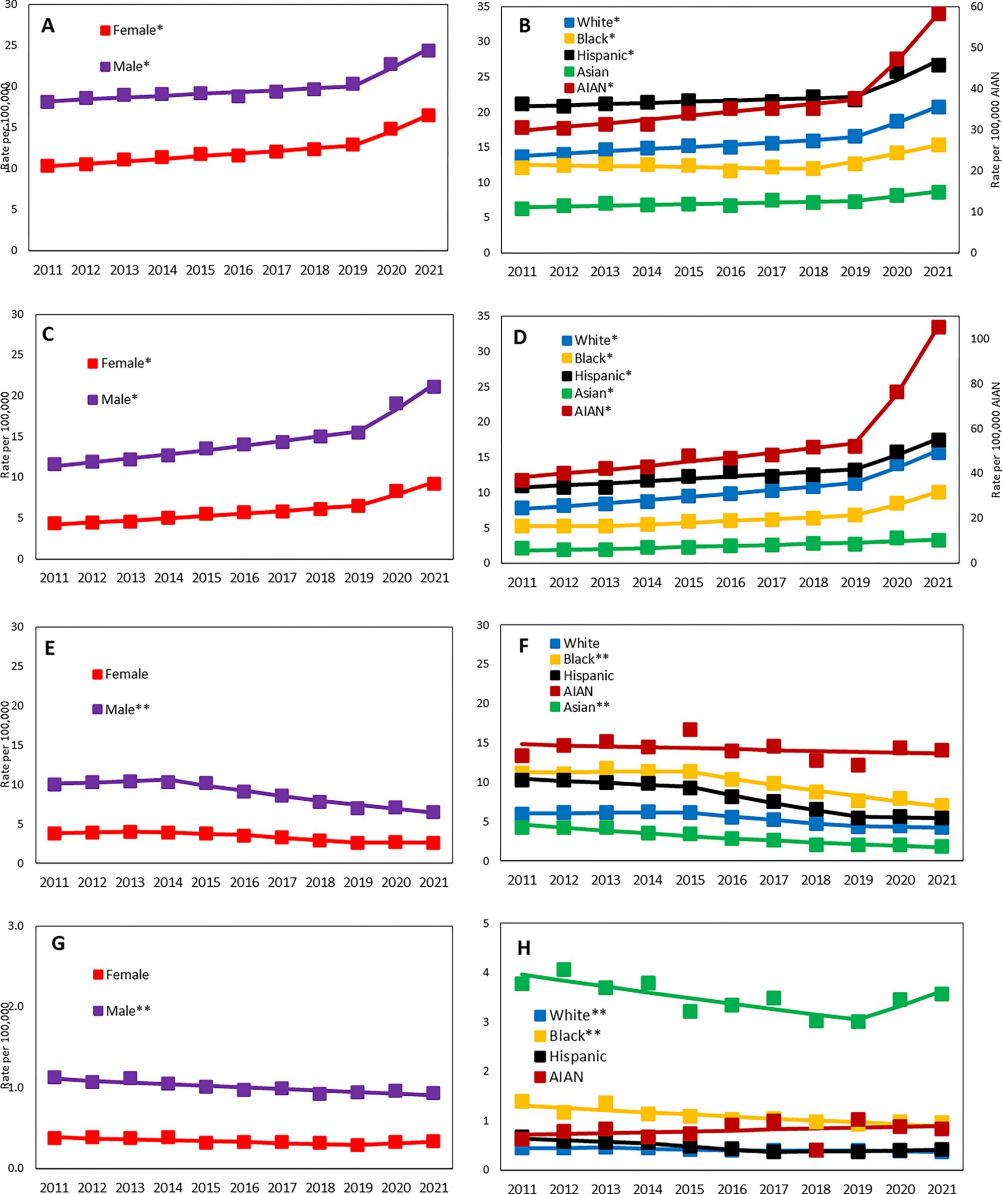
Trends in age-standardized CLD-related death rate, by sex and race/ethnicity: United States, 2011–2021. (A) NAFLD-related Death by Sex, (B) NAFLD-related Death by Race, (C) ALD-related Death by Sex, (D) ALD-related Death by Race, (E) HCV-related Death by Sex, (F) HCV-related Death by Race, (G) HBV-related Death by Sex, and (H) HBV-related Death by Race. * Significant increasing during the pandemic (2019–2021); p <0.05. ** Significant decreasing during the pandemic (2019–2021); p <0.05. NOTES: Data markers represent observed rates; lines are fitted rates based on joinpoint analysis. SOURCE: National Center for Health Statistics, National Vital Statistics System, Mortality.

Analysis of age-standardized death rates by sex showed that males and females experienced similar trends for NAFLD, HCV, HBV, and ALD during the entire study period although males had consistently higher all CLD-related death rates than females **(Table 2, Fig 3 and S8-S11 Tables in S1 File)**. However, during the 2019–2021, the annual increases in age-standardized death rates for NAFLD and ALD were higher in females versus those in males (NAFLD: AAPC = +13.60% [8.61% to 18.81%] vs. AAPC = +10.79% [6.28% to 15.49%] and ALD: AAPC = +19.77% [12.52% to 27.48%] vs. AAPC = +17.08% [13.39% to 20.89%]).

**Table 2:** Trends in age-standardized CLD-related death rates (per 100,000 population): Unites States, 2011–2021.

		Counts (Rate per 100,000)			2011–2021	2011–2019	2019–2021
Sex	Group	2011	2019	2020	AAPC (95%CI)	AAPC (95%CI)	AAPC (95%CI)
Both	NAFLD	34,465 (13.93)	47,330 (16.32)	59,230 (20.16)	3.8 (3.06–4.55)	1.82 (1.29–2.35)	12.13 (7.76–16.68)
	ALD	19,122 (7.76)	28,959 (10.74)	40,671 (14.95)	7.16 (6.39–7.93)	4.54 (3.97–5.11)	18.3 (13.76–23.03)
	HCV	17,717 (6.83)	14,313 (4.72)	13,950 (4.49)	-4.11 (-5.4 - -2.8)	-4.48 (-5.66 - -3.29)	-2.6 (-9.66–5)
	HBV	1,803 (0.72)	1,671 (0.59)	1,756 (0.61)	-1.73 (-3.36 - -0.07)	-2.68 (-3.69 - -1.66)	2.15 (-7.19–12.44)
	Female						
Female	NAFLD	13,681 (10.3)	20,136 (12.9)	25,745 (16.45)	4.84 (3.99–5.7)	2.76 (2.14–3.38)	13.6 (8.61–18.81)
	ALD	5,429 (4.34)	8,697 (6.43)	12,470 (9.18)	8.32 (7.08–9.58)	5.64 (4.69–6.59)	19.77 (12.52–27.48)
	HCV	5,068 (3.79)	4,030 (2.61)	4,105 (2.61)	-3.74 (-5.25 - -2.2)	-4.47 (-5.85 - -3.07)	-0.73 (-9.06–8.36)
	HBV	483 (0.37)	416 (0.28)	489 (0.33)	-1.57 (-4.89–1.86)	-3.41 (-5.45 - -1.31)	6.12 (-12.76–29.08)
	Male						
Male	NAFLD	20,784 (18.08)	27,194 (20.26)	33,485 (24.36)	3.04 (2.28–3.82)	1.19 (0.66–1.73)	10.79 (6.28–15.49)
	ALD	13,693 (11.54)	20,262 (15.47)	28,201 (21.11)	6.54 (5.92–7.17)	4.06 (3.6–4.52)	17.08 (13.39–20.89)
	HCV	12,649 (10.09)	10,283 (7.05)	9,845 (6.52)	-4.39 (-5.79 - -2.98)	-3.77 (-5.38 - -2.13)	-6.85 (-8.19 - -5.49)
	HBV	1,320 (1.12)	1,255 (0.93)	1,267 (0.92)	-1.99 (-2.59 - -1.39)	-1.99 (-2.59 - -1.39)	-1.99 (-2.59 - -1.39)

Abbreviation: AAPC, weighted average annual percent change; HCC, Hepatocellular carcinoma; CI, confidence interval.

Standardized to the 2000 US standard population by 10-year age group.

AAPC was a weighted average of annual percentage change with weights equal to the length of the detected time segments from the model selected by Joinpoint regression analysis.

During the 2019–2021, significant and substantial inclines in the age-standardized NAFLD- and ALD-related death were observed across race/ethnicity with the steepest increase in AIAN (AAPC = +25.22% [17.29% to 33.70%]) and in non-Hispanic White (AAPC = +12.55% [8.03% to 17.25%]) for NAFLD and those in AIAN (AAPC = +40.65% [30.71% to 51.35%]) and in non-Hispanic Black (AAPC = +22.36% [13.78% to 31.58%]) for ALD.

The steepest increases in the age-standardized NAFLD-related death rate were observed among AIAN female (AAPC = +25.38% [6.62% to 47.42%]) and AIAN male (AAPC = +25.09% [3.10% to 51.78%]), followed by non-Hispanic white female (AAPC = +14.28% [9.52% to 19.24%]), Hispanic female (AAPC = +11.30% [4.98% to 18.00%]), and Hispanic male (AAPC = +10.40% [3.49% to 17.77%]), whereas those in age-standardized ALD-relate death rate were observed among AIAN female (AAPC = +40.92% [28.67% to 54.33%]) and AIAN male (AAPC = +39.99% [25.15% to 56.59%]), followed by non-Hispanic black female (AAPC = +26.79% [9.72% to 46.51%]), non-Hispanic black male (AAPC = +19.40% [11.60% to 27.74%]), non-Hispanic white female (AAPC = +19.05% [13.36% to 25.02%]), and Hispanic Male (AAPC = +18.68% [0.11% to 40.70%]). Of note, the age-standardized CLD-related death rates increased more slowly or did not increase in Asian when compared with other races.

### Contribution of cause-specific death changes in decedents with CLD-related deaths after the pandemic start

**Fig 4:**
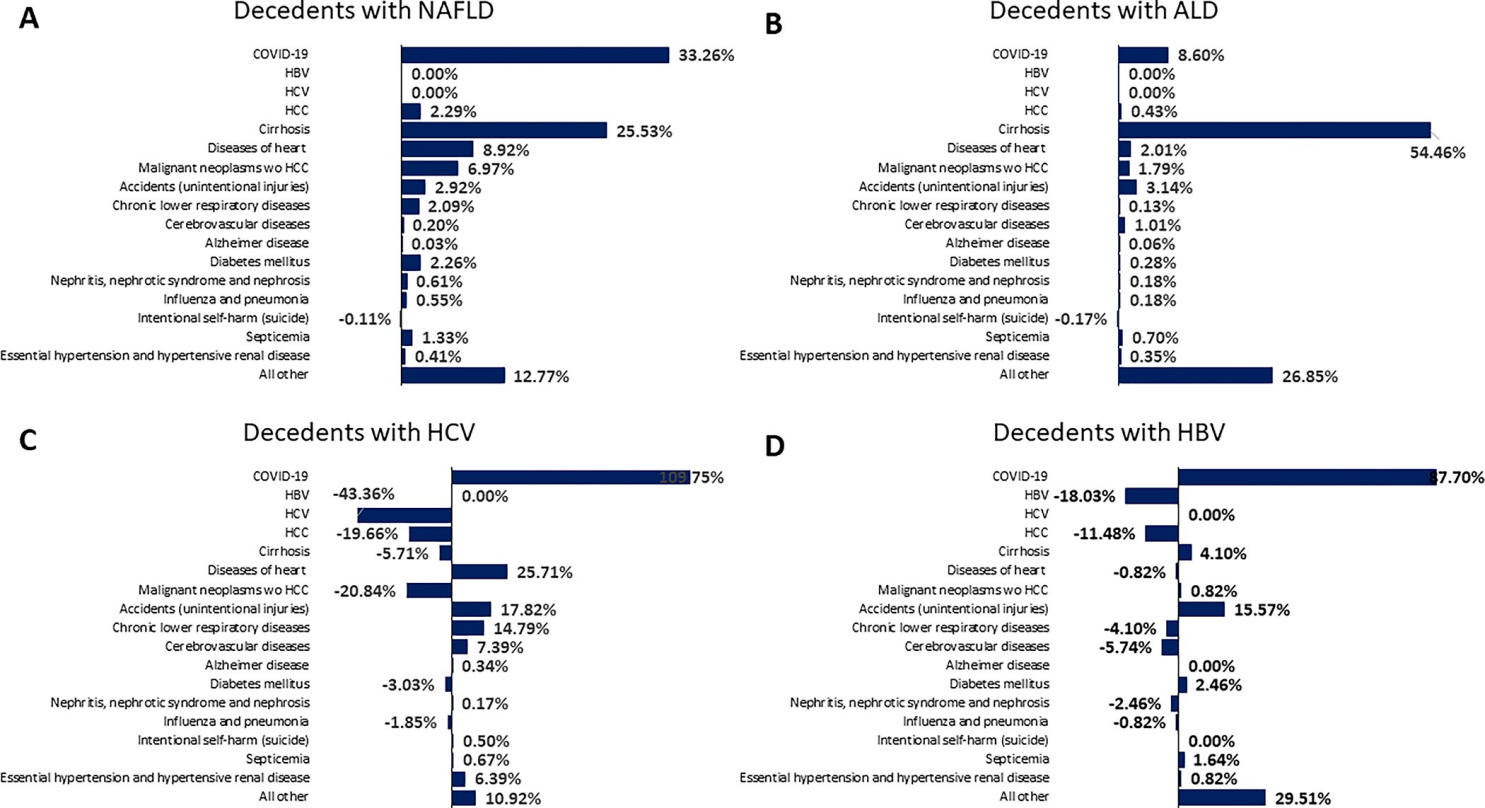
Change in the number of decedents with CLD attributable to various causes of death: United States, 2019–2020. (A) Decedents with NAFLD, (B) Decedents with ALD, (C) Decedents with HCV, and (D) Decedents with HBV. NOTES: Decedents with CLD were defined as having a CLD code for any cause of deaths. SOURCE: National Center for Health Statistics, National Vital Statistics System, Mortality.

Between 2019 and 2020, the highest increase in the number of decedents with CLD were observed for ALD (+24.7% from 28,959 to 36,118), followed by NAFLD (+15.0% from 47,330 to 54,409); HBV (+5.4% from 1,671 to 1,761)); and HCV (+4.2% from 14,313 to 14,908). The 17 causes of deaths selected accounted for most of the deaths with NAFLD (≥75%), ALD (≥75%), HCV (≥89%), and HBV (≥87%) in both years. To analyze the drivers of change in the number of decedents with CLD by each cause after the pandemic start, differences in the number of decedents with CLD between 2019 and 2020 were decomposed into differences in the number of deaths for each cause in decedents with CLD between 2019 and 2020. The contribution of each cause of death to the change in decedents with CLD was calculated by dividing difference in deaths from each cause between 2019 and 2020 by difference in CLD-related deaths, leading that sum of contribution of each cause was equal to 100%. The contribution of each cause of death varied markedly by CLD etiology **(Fig 4)**. COVID-19 contributed the most to the increase in the number of decedents with HCV (+109.8%) or HBV (+87.7%). These exceptionally large increases in COVID-19 deaths resulted in increases for HCV or HBV even though there were large decreases in liver-specific deaths (-68.7% for HCV and -25.4%). For decedents with NAFLD, COVID-19 contributed 33.3% of the increase in deaths, followed by cirrhosis (25.5%), diseases of heart (8.9%) and extrahepatic cancer (6.9%). In contrast, 54.5% of the increases in the number of decedents with ALD could be attributed to cirrhosis with only 8.6% related to COVID-19.

## Discussion

In this analysis, we have shown that liver-specific mortality rate was slowly increasing prior to COVID-19 pandemic for about a decade (about 1% each year from 2011–2019). However, this change in liver-specific mortality rates increased substantially during the COVID-19 pandemic (2020 and 2021). In fact, in comparison to 2019, liver-specific mortality increased 9% in 2020 and a 16% in 2021. This change in liver-specific mortality rates was primarily driven by increases in the cirrhosis-specific mortality. On the other hand, HCC-specific deaths did not change in a similar fashion. However, this may change in time due to the lack of HCC screening during the pandemic such that a longer period of follow up may be necessary to determine the impact of the pandemic on HCC [28].

In addition, our analysis demonstrated that the ALD and NAFLD were the main CLD etiologies that contributed to the increase in liver-specific mortality. Interestingly, among the decedents with ALD, cirrhosis accounted for 55% of deaths while COVID-19 infection accounted for less than 10%. This is consistent with the data that during the pandemic alcohol consumption may have led to more severe liver disease and effected the elderly more often than those younger [10, 11, 29, 30]. Additionally, our study findings validate the negative indirect effects of COVID-19 among those with ALD to include the hastening of cirrhosis [4, 7, 31–33].

The increase in NAFLD- related deaths has been noted previously in hospitalized patients [33]. However, our data provide a national perspective about the impact of the pandemic on NAFLD-related mortality. First, among those who died from cirrhosis (cirrhosis- specific deaths) during the study period, NAFLD accounted for 45% of these deaths as compared to 38% from ALD. In addition, among those who died with NAFLD, 42% died with cirrhosis as the leading cause of death as compared to only 10% for diseases of the heart. However, this finding maybe due to the way we defined NAFLD and may have led to an overestimation of cirrhosis among those with NAFLD because, in general, cardiac deaths are the leading cause of death among NAFLD [5, 6, 21, 33, 34]. When we investigated the contribution of COVID-19 in the increase of deaths among those with NAFLD, we found that COVID-19 was responsible for 33% of the increase in deaths from 2019–2020 followed by cirrhosis which explained 25% of the increase. The negative impact of COVID-19 among NAFLD has been reported in prior studies which showed that NAFLD’s risk factors increased the risk for COVID-19 related mortality which is now clearly seen on the national level [8, 9, 35–38]. The presence of fibrosis especially advanced fibrosis was also shown as a risk factor for increased mortality among those with NAFLD and COVID-19, findings validated here [39].

On the other hand, deaths among those with viral hepatitis (HBV and HCV) were decreasing from 2011–2019, a finding that is most likely due to the advent of and uptake of better viral suppression medication for HBV and curative medications, direct antiviral agents (DAAs), for HCV [40]. However, despite these encouraging downward trends prior to the pandemic, during the COVID-19 pandemic these trends stabilized. One reason for the stabilization could be due to the leveling of the HCC-specific mortality of which HBV and HCV are the main HCC contributors both of which stabilized. Another reason is most likely due to HBV infection has not been found to carry an additional risk for mortality during COVID-19 as in this study COVID-19 was the main contributor to the increase death rate among those with HBV not HBV or HCC. This finding also helps validate a recent study conducted in Hong Kong where among those with HBV during the pandemic, investigators determined that a current or past HBV infection was not associated with increased liver injury or mortality during COVID-19 [41]. Similar findings were noted for those with HCV. None the less, these trends need to be monitored closely in the coming years as recent reports suggest the diagnosis and prescribing of DAA’s decreased during the pandemic while at the same time the CDC announced more incident cases of HCV as a result of injection drug use and disruption of HCV public health services during the pandemic [42–44].

We also assessed the association of liver mortality during the COVID-19 pandemic according to sex and ethnicity. Overall, death rates were higher for males than females but during the pandemic females experienced a faster increase in their death rates compared to males driven by NAFLD and ALD for both. On closer inspection, both AIAN males and females, white males and females, black females, and Hispanic males were noted to have the fastest increases in liver deaths over the study time frame. However, when AIAN’s were excluded, Black females had the fastest annual increase in liver deaths driven by large increases in ALD and NAFLD related deaths compared to Black males [45, 46]

These findings fall in line with what has been reported overall as to who were most negatively affected from the COVID-19 pandemic [32–34]. None the less, the disparate findings of the significant negative effect of the pandemic among Black females require more investigation. One study using EHR record data reported that Blacks carried a higher proportion of alcohol associated gastrointestinal disorders during the first year of the pandemic than did other ethnicities [47], results that others have shown as well [48, 49]. It is known that alcohol use disorder was already increasing prior to the pandemic and during the first year of the pandemic alcohol sales increased most likely as a coping mechanism in response to the stress of the pandemic [4, 29, 47–49]. However, disturbing is that investigators determined the highest relative increase in alcohol use was among women and Black individuals with the highest relative increase for alcohol hepatitis among women and Blacks and the highest relative increase in ALD mortality was among women [49]. Here we have expanded these reported findings to indicate that it was Black females who carried the highest burden of ALD during the pandemic. These disparities may reflect inequitable access to treatment as well as social and economic exclusion. Our results will help healthcare workers and policy decision makers develop future national and global health emergency care plans out lining treatment and outreach protocols for those with CLD who may be most vulnerable (females, AIAN, Black, have NAFLD, ALD, cirrhosis, alcohol use disorder, depression and or multiple comorbidities) for adverse outcomes.

Our study is not free of limitations. The causes of death and mortality data were obtained from death certificates which may be subject to misclassification, underestimation, and unmeasured factors, which are important clinical considerations. For this reason, liver deaths were identified as either an underlying cause of death or as a liver complication noted as an underlying or contributory cause of death. Similarly, any mention of CLD as an underlying or contributory cause of death were defined as CLD-related deaths. Nevertheless, given the possibility for these coding errors in both directions, we feel that over time the miscoding balanced out without a net gain or loss of the pertinent data. Also, our extended definition of NAFLD including the ICD-codes for cryptogenic cirrhosis may have led to an overestimation. However, NAFLD defined as the ICD 10 codes of K76.0 and K75.81 certainly underestimates the true prevalence of NAFLD. Therefore, we believe that our estimates of NAFLD in the United States reflect the true burden of this liver disease [17]. Finally, there may be other unfounded contributory factors which were unable to control for. NVSS lacks granular data such as radiographic, physiological, and laboratory parameters, which are important clinical considerations. However, our goal was to evaluate the impact of the COVID-19 pandemic on liver-related deaths by trend-analysis which we were able to do using joinpoint trend analysis.

## Conclusion

In summary, our analysis provides data about liver-specific and liver-related mortality in the United States prior to and during the COVID-19 pandemic. We found that during the COVID-19 pandemic (2020–2021), there was an increase in liver-specific, especially cirrhosis-specific mortality. NAFLD and ALD were the main etiologic drivers of this increase whereas HBV and HCV deaths stabilized during the pandemic. However, the main contributor to the increase in deaths differed by liver disease etiology. The increase in ALD related deaths during the pandemic was due to an increase in cirrhosis-specific deaths accounting for 1 in 2 deaths while COVID-19 was the main contributor to deaths among those with NAFLD accounting for 1 in 3 deaths although cirrhosis did account for 1 in 4 deaths for this group. COVID-19 was the main contributor for increased deaths among those with viral hepatitis not the presence of liver disease. In addition, we determined that among the decedents with CLD, females, Blacks, AIANs, those with cirrhosis, and those with multi-comorbidities were the most negatively affected during the pandemic. These noted disparities suggest that both healthcare workers and policy makers need to incorporate outreach strategies in addition to medical strategies in future preparedness plans to ensure all people with CLD have access to continual, uninterrupted care.

## Supporting information

S1 File (DOCX)

## List of abbreviations

GBD: global burden of disease
NAFLD: non-alcoholic fatty liver disease
HBV: hepatitis B virus
HCV: hepatitis C virus
ALD: alcohol liver disease
CLD: chronic liver disease
