# Supplementary material for: Changes in mortality due to Chronic Liver Diseases (CLD) during the COVID-19 pandemic: Data from the United States’ National Vital Statistics System
Source: PLoS One. 2024 Sep 3;19(9):e0289202. doi: 10.1371/journal.pone.0289202 (PMC11371215; doi:10.1371/journal.pone.0289202)
Supplement: S1 File — (DOCX) [file pone.0289202.s001.docx]

| **Supplementary Table 1. List of ICD 10 codes for Cause-specific Deaths** |  |
| --- | --- |
|  | ICD 10 CODE |
| **Liver disease-related diagnosis** | |
| Acute and subacute necrosis of liver | K72.0x, K76.2 |
| Chronic liver disease | K70.xx, K72.1x, K73.x, K74.xx, K75.4, K75.8x, K76.0, K76.89, K76.9 |
| Cirrhosis | K65.2, K70.11, K70.2, K70.3, K70.4, K71.51, K71.7, K72.xx, K74.x, K76.6, K76.7, I85, R18 |
| Liver abscess and sequelae of chronic liver disease | K72.9x, K75.0-1, K76.6-7 |
| Malignant neoplasm of liver and intrahepatic bile duct | C22.0-2, C22.7-9 |
| Other disorders of the liver | K71.0-1x, K71.3-9, K72, K75, K75.3, K75.9, K76.1, K76.8x, K76.9, K77 |
| Viral hepatitis | B15.x - B19.xx |
| **Liver disease-related Complications** | |
| Acute kidney injury | N17.9, N28.9 |
| Ascites | K70.11, K70.31, K71.51, R18.x |
| Cellulitis | K12.2, L03.01x, L03.03x, L03.11x, L03.211, L03.213, L03.221, L03.31x, L03.81x, L03.90 |
| Cholangitis | K83.0x |
| Encephalopathy | G93.4x, K72.xx |
| Esophageal varices | I85.xx |
| Gastrointestinal hemorrhage | K92.0-2 |
| Hepatorenal syndrome | K76.7 |
| Hydrothorax | J90, J94.8 |
| Hyponatremia | E87.1 |
| Infection (including sepsis, pneumonia) | A04.xx, A08.xx, A09, A40.x, A41.x, A48.1, A48.3, A48.8, A49.0x, A49.1, A49.8-9, B25.0, B44.0, B44.1, B49, B95.xx, B96.xx, J10.0x, J11.0x, J12.xx, J13, J14, J15.xx, J16.x, J17, J18.0-1, J18.9 |
| Jaundice | R17 |
| Liver failure | K70.4x, K72.xx |
| Peritonitis | K65.x, K67, K68.12, K68.19, K68.9 |
| Portal hypertension | K76.6 |
| Portal vein thrombosis or deep vein thrombosis | I80.1x - I80.9, I81, I82.0 - I82.70x, I82.72x, I82.Axx - I82.Cxx, I82.89x, I82.9x |
| Renal failure | N18.x, N19, R94.4 |
| Sepsis | A40.x, A41.x, R65.2x |
| Volume overload or dehydration | E86.0, E87.7x, R60.x |

| S Table 2. The 10 Leading Causes: United States, 2019, 2020, and 2021 | | | | | | | | | |
| --- | --- | --- | --- | --- | --- | --- | --- | --- | --- |
| Underlying Causes of Deaths | 2019 | | | 2020 | | | 2021 | | |
|  | Rank* | Deaths | Percent of total deaths | Rank* | Deaths | Percent of total deaths | Rank* | Deaths | Percent of total deaths |
| All-causes |  | 2,820,654 |  |  | 3,348,877 |  |  | 3,428,561 |  |
| Diseases of heart (I00–I09,I11,I13,I20–I51) | 1 | 659,804 | 23.4% | 1 | 697,384 | 20.8% | 1 | 695,985 | 20.30% |
| Extrahepatic cancer (C00–C97) | 2 | 598,782 | 21.2% | 2 | 601,373 | 18.0% | 2 | 592,173 | 17.27% |
| COVID-19 (U07. 1) |  |  | 0.0% | 3 | 351,307 | 10.5% | 3 | 417,252 | 12.17% |
| Accidents (unintentional injuries) (V01–X59,Y85–Y86) | 3 | 166,899 | 5.9% | 4 | 193,767 | 5.8% | 4 | 217,395 | 6.34% |
| Chronic lower respiratory diseases (J40–J47) | 4 | 156,859 | 5.6% | 7 | 152,511 | 4.6% | 6 | 142,251 | 4.15% |
| Cerebrovascular diseases (I60–I69) | 5 | 150,007 | 5.3% | 5 | 160,206 | 4.8% | 5 | 162,802 | 4.75% |
| Alzheimer disease (G30) | 6 | 121,539 | 4.3% | 6 | 134,287 | 4.0% | 7 | 119,442 | 3.48% |
| Liver disease** | 7 | 95,380 | 3.4% | 8 | 103,795 | 3.1% | 8 | 110,534 | 3.22% |
| Diabetes mellitus (E10–E14) | 8 | 87,708 | 3.1% | 9 | 102,140 | 3.0% | 9 | 103,264 | 3.01% |
| Nephritis, nephrotic syndrome and nephrosis (N00–N07,N17–N19,N25–N27) | 9 | 51,522 | 1.8% | 10 | 52,517 | 1.6% | 10 | 54,355 | 1.59% |
| Influenza and pneumonia (J09–J18) | 10 | 49,432 | 1.8% | 11 | 53,280 | 1.6% | 12 | 41,738 | 1.22% |
| Intentional self-harm (suicide)(*U03,X60–X84,Y87.0) | 11 | 44,902 | 1.6% | 12 | 43,296 | 1.3% | 11 | 45,379 | 1.32% |
| * Based on number of deaths ** By using the published coding algorithm  SOURCE: NCHS, National Vital Statistics System, Mortality | | | | | | | | | |

| S Table 3. Individuals Aged +20 Years Died by Liver Disease, HCC, and Cirrhosis Reported on Death Certificates in the United States, 2011-2021 | | | |  |
| --- | --- | --- | --- | --- |
|  | Liver Deaths (n=1,012,372) | HCC deaths (n=117,752) | Cirrhosis Deaths (n=442,001) |  |
| Age ,y mean ± SD | 63.73 ±13.06 | 67.18 ±10.74 | 62.01 ±12.89 |  |
| aged 20-44 | 1120 (0.11%) | 109 (0.09%) | 284 (0.06%) |  |
| aged 45-64 | 453897 (44.84%) | 45004 (38.22%) | 217573 (49.22%) |  |
| aged 65-84 | 421927 (41.68%) | 61806 (52.49%) | 167352 (37.86%) |  |
| aged ≥ 85 | 74939 (7.40%) | 9393 (7.98%) | 24797 (5.61%) |  |
| Male | 636949 (62.92%) | 91380 (77.60%) | 276899 (62.65%) |  |
| Race |  |  |  |  |
| non-Hispanic white | 710451 (70.18%) | 72971 (61.97%) | 319930 (72.38%) |  |
| non-Hispanic black | 105470 (10.42%) | 18095 (15.37%) | 36513 (8.26%) |  |
| Hispanic | 140373 (13.87%) | 16642 (14.13%) | 66498 (15.04%) |  |
| non-Hispanic AIAN | 32824 (3.24%) | 8266 (7.02%) | 7610 (1.72%) |  |
| non-Hispanic Asian | 19381 (1.91%) | 1320 (1.12%) | 9722 (2.20%) |  |
| Other Race | 3873 (0.38%) | 458 (0.39%) | 1728 (0.39%) |  |
| Education |  |  |  |  |
| below college | 135249 (13.86%) | 16316 (14.41%) | 58072 (13.62%) |  |
| College | 653671 (67.00%) | 76610 (67.67%) | 288671 (67.70%) |  |
| Above college | 186707 (19.14%) | 20280 (17.91%) | 79671 (18.68%) |  |
| Marital status |  |  |  |  |
| Married | 431725 (43.20%) | 58580 (50.41%) | 176092 (40.39%) |  |
| Widow | 150433 (15.05%) | 16518 (14.21%) | 60944 (13.98%) |  |
| Single | 165112 (16.52%) | 15537 (13.37%) | 78500 (18.00%) |  |
| Divorced | 252086 (25.22%) | 25580 (22.01%) | 120488 (27.63%) |  |
| Comorbidities* |  |  |  |  |
| COVID | 2072 (0.20%) | 176 (0.15%) | 1099 (0.25%) |  |
| HBV | 12303 (1.22%) | 3281 (2.79%) | 1473 (0.33%) |  |
| HCV | 123498 (12.20%) | 26308 (22.34%) | 19627 (4.44%) |  |
| NAFLD | 317965 (31.41%) | 14673 (12.46%) | 200113 (45.27%) |  |
| ALD | 244146 (24.12%) | 4719 (4.01%) | 168104 (38.03%) |  |
| Elixhauser Comorbidities* |  |  |  |  |
| Congestive heart failure | 46207 (4.56%) | 3023 (2.57%) | 22868 (5.17%) |  |
| Cardiac arrhythmias | 26797 (2.65%) | 2377 (2.02%) | 12031 (2.72%) |  |
| Valvular disease | 5572 (0.55%) | 430 (0.37%) | 2393 (0.54%) |  |
| Pulmonary circulation Disorders | 9976 (0.99%) | 1164 (0.99%) | 3917 (0.89%) |  |
| Peripheral vascular disorders | 8038 (0.79%) | 729 (0.62%) | 3516 (0.80%) |  |
| Hypertension, uncomplicated | 57944 (5.72%) | 8115 (6.89%) | 23418 (5.30%) |  |
| Hypertension, complicated | 12623 (1.25%) | 1083 (0.92%) | 6124 (1.39%) |  |
| Paralysis | 713 (0.07%) | 98 (0.08%) | 259 (0.06%) |  |
| Other neurological disorders | 24881 (2.46%) | 1319 (1.12%) | 12258 (2.77%) |  |
| Chronic pulmonary disease | 40667 (4.02%) | 4690 (3.98%) | 18332 (4.15%) |  |
| Diabetes, uncomplicated | 55862 (5.52%) | 8340 (7.08%) | 22294 (5.04%) |  |
| Diabetes, complicated | 12494 (1.23%) | 1350 (1.15%) | 5542 (1.25%) |  |
| Hypothyroidism | 5072 (0.50%) | 496 (0.42%) | 2225 (0.50%) |  |
| Renal failure | 87870 (8.68%) | 4762 (4.04%) | 41308 (9.35%) |  |
| Liver disease | 795218 (78.55%) | 54260 (46.08%) | 441897 (99.98%) |  |
| Peptic ulcer disease excluding bleeding | 1312 (0.13%) | 99 (0.08%) | 716 (0.16%) |  |
| AIDS/H1V | 1797 (0.18%) | 602 (0.51%) | 715 (0.16%) |  |
| Lymphoma | 2598 (0.26%) | 307 (0.26%) | 1115 (0.25%) |  |
| Metastatic cancer | 24795 (2.45%) | 8000 (6.79%) | 1575 (0.36%) |  |
| Solid tumor without metastasis | 307114 (30.34%) | 117752 (100.00%) | 11698 (2.65%) |  |
| Rheumatoid arthritis/ collagen vascular diseases | 3109 (0.31%) | 193 (0.16%) | 1476 (0.33%) |  |
| Coagulopathy | 31984 (3.16%) | 1430 (1.21%) | 17564 (3.97%) |  |
| Obesity | 8768 (0.87%) | 426 (0.36%) | 3880 (0.88%) |  |
| Weight loss | 16817 (1.66%) | 1555 (1.32%) | 7956 (1.80%) |  |
| Fluid and electrolyte disorders | 27042 (2.67%) | 1856 (1.58%) | 12671 (2.87%) |  |
| Blood loss anemia | 944 (0.09%) | 51 (0.04%) | 579 (0.13%) |  |
| Deficiency anemia | 801 (0.08%) | 77 (0.07%) | 413 (0.09%) |  |
| Alcohol abuse | 248640 (24.56%) | 9121 (7.75%) | 181291 (41.02%) |  |
| Drug abuse | 8336 (0.82%) | 896 (0.76%) | 2810 (0.64%) |  |
| Psychoses | 1342 (0.13%) | 156 (0.13%) | 516 (0.12%) |  |
| Depression | 4225 (0.42%) | 305 (0.26%) | 2030 (0.46%) |  |
| Elixhauser comorbidity index (ECI), mean ± SD | 1.86 ±1.01 | 2.00 ±1.08 | 1.96 ±0.99 |  |
| Number of Comorbidity = 0 | 4904 (0.48%) |  | 102 (0.02%) |  |
| Number of Comorbidity = 1 | 446674 (44.12%) | 47267 (40.14%) | 164069 (37.12%) |  |
| Number of Comorbidity = 2 | 348709 (34.44%) | 39499 (33.54%) | 178448 (40.37%) |  |
| Number of Comorbidity ≥ 3 | 212085 (20.95%) | 30986 (26.31%) | 99382 (22.48%) |  |
| Abbreviation: SD, Standard deviation; HCC, Hepatocellular carcinoma. * Defined as an underlying or contributory cause of death | | | |  |
|  |  |  |  |  |
|  |  |  |  |  |

| **S Table 4. Trends in Age-standardized Liver Death* Rate per 100,000 population by Sex and Race/Ethnicity in the Unites States, 2011-2021** | | | | | | | |
| --- | --- | --- | --- | --- | --- | --- | --- |
|  |  | Counts (Rate per 100,000) | | | 2011-2021 | 2011-2019 | 2019-2021 |
| Sex | Group | 2011 | 2019 | 2021 | AAPC (95%CI) | AAPC (95%CI) | AAPC (95%CI) |
| Both | All Race | 78,468 (31.51) | 95,380 (33.3) | 110,534 (38.31) | 1.99 (1.55 - 2.42) | 0.66 (0.25 - 1.08) | 7.46 (5.10 - 9.86) |
|  | non-Hispanic white | 55,522 (29.97) | 67,038 (33.12) | 76,779 (38.72) | 2.62 (2.06 - 3.19) | 1.21 (0.68 - 1.74) | 8.49 (5.39 - 11.67) |
|  | non-Hispanic black | 8,653 (32.41) | 9,609 (30.07) | 10,687 (33.01) | 0.21 (-0.66 - 1.07) | -0.85 (-1.7 - 0) | 4.55 (0.17 - 9.11) |
|  | Hispanic | 10,300 (44.92) | 13,449 (42.12) | 16,242 (46.54) | 0.33 (-0.35 - 1.01) | -0.78 (-1.45 - -0.11) | 4.89 (1.49 - 8.39) |
|  | non-Hispanic AIAN | 1,290 (77.98) | 1,789 (91.54) | 2,691 (152.06) | 6.71 (4.47 - 8.99) | 1.96 (-0.31 - 4.27) | 28.04 (16.88 - 40.27) |
|  | non-Hispanic Asian | 2,509 (24.45) | 3,190 (21.15) | 3,497 (22.51) | -1.11 (-2.12 - -0.09) | -2.16 (-2.85 - -1.47) | 3.21 (-2.57 - 9.33) |
| Female | All Race | 28,220 (21.31) | 36,037 (23.75) | 42,499 (28.12) | 2.87 (2.6 - 3.13) | 1.34 (1.08 - 1.60) | 9.19 (7.81 - 10.59) |
|  | non-Hispanic white | 20,141 (20.56) | 25,707 (24.29) | 29,953 (29.21) | 3.66 (3.24 - 4.09) | 2.13 (1.72 - 2.54) | 10.06 (7.86 - 12.3) |
|  | non-Hispanic black | 3,147 (21.65) | 3,561 (20.39) | 4,170 (23.92) | 1.19 (-0.33 - 2.73) | -0.56 (-2.02 - 0.93) | 8.48 (0.61 - 16.96) |
|  | Hispanic | 3,393 (29.45) | 4,705 (28.66) | 5,564 (31.73) | 0.77 (-0.36 - 1.91) | -0.38 (-1.48 - 0.73) | 5.51 (-0.26 - 11.63) |
|  | non-Hispanic AIAN | 592 (68.65) | 843 (82.57) | 1,232 (134.42) | 6.75 (6 - 7.5) | 1.89 (1.14 - 2.65) | 28.6 (24.76 - 32.56) |
|  | non-Hispanic Asian | 905 (16.65) | 1,150 (13.84) | 1,335 (15.78) | -1.03 (-2.49 - 0.46) | -2.65 (-3.64 - -1.64) | 5.73 (-2.72 - 14.91) |
| Male | All Race | 50,248 (42.99) | 59,343 (44.1) | 68,035 (49.53) | 1.45 (0.79 - 2.12) | 0.33 (-0.32 - 1) | 6.04 (2.7 - 9.48) |
|  | non-Hispanic white | 35,381 (40.4) | 41,331 (42.89) | 46,826 (49.01) | 1.99 (1.17 - 2.82) | 0.72 (-0.08 - 1.53) | 7.24 (3.06 - 11.59) |
|  | non-Hispanic black | 5,506 (46.07) | 6,048 (42.8) | 6,517 (44.84) | -0.33 (-1.39 - 0.75) | -0.98 (-2.02 - 0.07) | 2.33 (-3.03 - 7.98) |
|  | Hispanic | 6,907 (62.19) | 8,744 (57.15) | 10,678 (62.17) | -0.03 (-0.59 - 0.53) | -1.02 (-1.62 - -0.41) | 4.02 (1.72 - 6.38) |
|  | non-Hispanic AIAN | 698 (88.34) | 946 (101.69) | 1,459 (170.48) | 6.59 (3.48 - 9.8) | 1.74 (-1.33 - 4.9) | 28.43 (12.14 - 47.09) |
|  | non-Hispanic Asian | 1,604 (33.97) | 2,040 (30.2) | 2,162 (30.55) | -1.09 (-2.82 - 0.66) | -1.3 (-3.39 - 0.84) | -0.26 (-1.79 - 1.3) |
| Abbreviation: AAPC, weighted average annual percent change; AIAN, American Indian or Alaska Native; CI, confidence interval. Standardized to the 2000 US standard population by 10-year age group. AAPC was a weighted average of annual percentage change with weights equal to the length of the detected time segments from the model selected by Joinpoint regression analysis. | | | | | | | |

| **S Table 5. Trends in Age-standardized Cirrhosis Death* Rate per 100,000 population by Sex and Race/Ethnicity in the Unites States, 2011-2021** | | | | | | | |
| --- | --- | --- | --- | --- | --- | --- | --- |
|  |  | Counts (Rate per 100,000) | | | 2011-2021 | 2011-2019 | 2019-2021 |
| Sex | Group | 2011 | 2019 | 2021 | AAPC (95%CI) | AAPC (95%CI) | AAPC (95%CI) |
| Both | All Race | 32,286 (13.02) | 42,795 (15.24) | 53,309 (18.86) | 3.75 (3.18 - 4.32) | 1.95 (1.56 - 2.35) | 11.25 (7.89 - 14.71) |
|  | non-Hispanic white | 23,844 (12.98) | 30,861 (15.71) | 37,661 (19.65) | 4.23 (3.66 - 4.8) | 2.38 (1.99 - 2.77) | 11.95 (8.57 - 15.44) |
|  | non-Hispanic black | 2,782 (10.23) | 3,465 (10.81) | 4,303 (13.19) | 2.62 (1.69 - 3.56) | 0.64 (-0.01 - 1.3) | 10.92 (5.42 - 16.71) |
|  | Hispanic | 4,501 (19.22) | 6,615 (20.09) | 8,602 (23.73) | 2.23 (1.23 - 3.25) | 0.64 (-0.37 - 1.66) | 8.86 (3.97 - 13.98) |
|  | non-Hispanic AIAN | 602 (35.68) | 928 (47.88) | 1,511 (85.98) | 8.54 (6.97 - 10.14) | 3.38 (2.2 - 4.58) | 31.89 (21.92 - 42.69) |
|  | non-Hispanic Asian | 485 (4.68) | 798 (5.25) | 917 (5.89) | 2.19 (0.34 - 4.08) | 1.41 (-0.11 - 2.96) | 5.37 (-0.85 - 11.97) |
| Female | All Race | 11,948 (9.1) | 16,139 (10.87) | 20,245 (13.74) | 4.26 (3.43 - 5.11) | 2.39 (1.8 - 2.98) | 12.11 (7.2 - 17.24) |
|  | non-Hispanic white | 8,929 (9.23) | 11,878 (11.64) | 14,577 (14.79) | 4.97 (4.1 - 5.84) | 3.15 (2.56 - 3.75) | 12.55 (7.47 - 17.88) |
|  | non-Hispanic black | 1,079 (7.28) | 1,363 (7.78) | 1,780 (10.29) | 3.79 (1.85 - 5.76) | 1.12 (-0.24 - 2.5) | 15.16 (3.78 - 27.8) |
|  | Hispanic | 1,465 (12.62) | 2,127 (12.76) | 2,712 (15.12) | 1.65 (0.36 - 2.95) | -0.1 (-1.04 - 0.84) | 8.97 (1.61 - 16.87) |
|  | non-Hispanic AIAN | 282 (32.5) | 440 (43.18) | 697 (76.75) | 8.14 (6.84 - 9.47) | 2.96 (1.99 - 3.95) | 31.6 (23.23 - 40.54) |
|  | non-Hispanic Asian | 178 (3.28) | 302 (3.63) | 356 (4.22) | 1.65 (0.54 - 2.78) | 1.65 (0.54 - 2.78) | 1.65 (0.54 - 2.78) |
| Male | All Race | 20,338 (17.39) | 26,656 (20.08) | 33,064 (24.37) | 3.37 (2.71 - 4.04) | 1.71 (1.25 - 2.17) | 10.33 (6.46 - 14.33) |
|  | non-Hispanic white | 14,915 (17.09) | 18,983 (20.16) | 23,084 (24.8) | 3.72 (2.91 - 4.54) | 1.92 (1.37 - 2.48) | 11.25 (6.46 - 16.25) |
|  | non-Hispanic black | 1,703 (13.96) | 2,102 (14.68) | 2,523 (16.8) | 1.78 (0.53 - 3.03) | 0.38 (-0.48 - 1.25) | 7.56 (0.45 - 15.18) |
|  | Hispanic | 3,036 (26.44) | 4,488 (28.03) | 5,890 (32.57) | 2.28 (1.35 - 3.21) | 1.01 (0.07 - 1.96) | 7.51 (3.07 - 12.13) |
|  | non-Hispanic AIAN | 320 (39.22) | 488 (52.94) | 814 (95.55) | 8.84 (5.47 - 12.31) | 3.72 (1.16 - 6.34) | 31.98 (11.5 - 56.21) |
|  | non-Hispanic Asian | 308 (6.29) | 497 (7.2) | 561 (7.8) | 2.29 (0.94 - 3.66) | 2.29 (0.94 - 3.66) | 2.29 (0.94 - 3.66) |
| Abbreviation: AAPC, weighted average annual percent change; AIAN, American Indian or Alaska Native; CI, confidence interval. Standardized to the 2000 US standard population by 10-year age group. AAPC was a weighted average of annual percentage change with weights equal to the length of the detected time segments from the model selected by Joinpoint regression analysis. | | | | | | | |

| **S Table 6. Trends in Age-standardized HCC Death* Rate per 100,000 population by Sex and Race/Ethnicity in the Unites States, 2011-2021** | | | | | | | |
| --- | --- | --- | --- | --- | --- | --- | --- |
|  |  | Counts (Rate per 100,000) | | | 2011-2021 | 2011-2019 | 2019-2021 |
| Sex | Group | 2011 | 2019 | 2021 | AAPC (95%CI) | AAPC (95%CI) | AAPC (95%CI) |
| Both | All Race | 8,997 (3.53) | 11,872 (3.86) | 12,122 (3.84) | 0.87 (0.17 - 1.57) | 1.18 (0.34 - 2.03) | -0.39 (-1.32 - 0.54) |
|  | non-Hispanic white | 5,574 (2.87) | 7,414 (3.25) | 7,634 (3.32) | 1.55 (0.72 - 2.4) | 1.75 (0.74 - 2.76) | 0.79 (-0.32 - 1.91) |
|  | non-Hispanic black | 1,439 (5.26) | 1,793 (5.39) | 1,667 (4.95) | -0.48 (-2.24 - 1.32) | 0.36 (-1.01 - 1.74) | -3.73 (-9.55 - 2.46) |
|  | Hispanic | 1,181 (5.53) | 1,695 (5.61) | 1,815 (5.57) | 0.53 (-0.68 - 1.75) | 0.53 (-0.68 - 1.75) | 0.53 (-0.68 - 1.75) |
|  | non-Hispanic AIAN | 79 (4.93) | 133 (6.42) | 136 (6.75) | 1.89 (0.1 - 3.71) | 1.89 (0.1 - 3.71) | 1.89 (0.1 - 3.71) |
|  | non-Hispanic Asian | 706 (6.7) | 790 (5.2) | 800 (5.08) | -2.76 (-4.1 - -1.41) | -2.77 (-4.36 - -1.15) | -2.74 (-4.56 - -0.89) |
| Female | All Race | 1,958 (1.43) | 2,706 (1.64) | 2,785 (1.67) | 1.96 (1.32 - 2.6) | 1.96 (1.32 - 2.6) | 1.96 (1.32 - 2.6) |
|  | non-Hispanic white | 1,164 (1.11) | 1,611 (1.32) | 1,716 (1.42) | 2.74 (2.03 - 3.45) | 2.74 (2.03 - 3.45) | 2.74 (2.03 - 3.45) |
|  | non-Hispanic black | 291 (1.99) | 412 (2.24) | 345 (1.86) | -0.68 (-4.85 - 3.68) | 1.34 (-1.44 - 4.19) | -8.35 (-28.19 - 16.96) |
|  | Hispanic | 293 (2.79) | 427 (2.75) | 445 (2.73) | 0.94 (-0.73 - 2.64) | 0.94 (-0.73 - 2.64) | 0.94 (-0.73 - 2.64) |
|  | non-Hispanic AIAN | 21 (2.49) | 44 (4.08) | 40 (3.81) | 1.06 (-2.29 - 4.52) | 1.06 (-2.29 - 4.52) | 1.06 (-2.29 - 4.52) |
|  | non-Hispanic Asian | 190 (3.46) | 209 (2.52) | 229 (2.71) | -3.01 (-5.84 - -0.1) | -3.46 (-6.85 - 0.06) | -1.21 (-2.93 - 0.55) |
| Male | All Race | 7,039 (5.96) | 9,166 (6.44) | 9,337 (6.35) | 0.62 (0.03 - 1.21) | 1 (0.29 - 1.72) | -0.91 (-1.69 - -0.12) |
|  | non-Hispanic white | 4,410 (4.89) | 5,803 (5.46) | 5,918 (5.48) | 1.14 (0.99 - 1.3) | 1.37 (1.18 - 1.55) | 0.26 (0.02 - 0.5) |
|  | non-Hispanic black | 1,149 (9.43) | 1,381 (9.55) | 1,323 (9.02) | -0.35 (-1.96 - 1.28) | 0.39 (-0.85 - 1.65) | -3.25 (-8.59 - 2.4) |
|  | Hispanic | 888 (8.76) | 1,269 (9.02) | 1,370 (8.83) | 0.31 (-0.93 - 1.56) | 0.31 (-0.93 - 1.56) | 0.31 (-0.93 - 1.56) |
|  | non-Hispanic AIAN | 60 (8.02) | 91 (9.49) | 98 (10.28) | 2.39 (0.82 - 3.98) | 2.39 (0.82 - 3.98) | 2.39 (0.82 - 3.98) |
|  | non-Hispanic Asian | 516 (10.74) | 582 (8.53) | 572 (7.97) | -2.37 (-3.13 - -1.61) | -2.37 (-3.13 - -1.61) | -2.37 (-3.13 - -1.61) |
| Abbreviation: AAPC, weighted average annual percent change; AIAN, American Indian or Alaska Native; CI, confidence interval. Standardized to the 2000 US standard population by 10-year age group. AAPC was a weighted average of annual percentage change with weights equal to the length of the detected time segments from the model selected by Joinpoint regression analysis. | | | | | | | |

| S Table 7. Decedents Aged +20 Years With NAFLD, ALD, HCV, and HBV Reported on Death Certificates in the United States, 2011-2021 | | | | |
| --- | --- | --- | --- | --- |
|  | NAFLD (n=479,011) | ALD (n=290,952) | HCV (n=189,398) | HBV (n=19,270) |
| Age ,y mean ± SD | 65.74 ±12.96 | 56.93 ±11.82 | 60.73 ±9.75 | 61.49 ±12.79 |
| aged 20-44 | 716 (0.15%) | 377 (0.13%) | 130 (0.07%) | 21 (0.11%) |
| aged 45-64 | 184552 (38.53%) | 170875 (58.73%) | 116522 (61.52%) | 9677 (50.22%) |
| aged 65-84 | 229593 (47.93%) | 78080 (26.84%) | 61335 (32.38%) | 7057 (36.62%) |
| aged ≥ 85 | 42519 (8.88%) | 3850 (1.32%) | 4483 (2.37%) | 988 (5.13%) |
| Male | 279469 (58.34%) | 204765 (70.38%) | 134973 (71.26%) | 14064 (72.98%) |
| Race |  |  |  |  |
| non-Hispanic white | 350438 (73.16%) | 206558 (70.99%) | 119494 (63.09%) | 8708 (45.19%) |
| non-Hispanic black | 41809 (8.73%) | 22107 (7.60%) | 34518 (18.23%) | 3581 (18.58%) |
| Hispanic | 67398 (14.07%) | 46014 (15.81%) | 26611 (14.05%) | 1510 (7.84%) |
| Asian | 10312 (2.15%) | 4027 (1.38%) | 4252 (2.25%) | 5199 (26.98%) |
| American Indian or Alaska Native | 7420 (1.55%) | 10817 (3.72%) | 3132 (1.65%) | 152 (0.79%) |
| Other Race | 1634 (0.34%) | 1429 (0.49%) | 1391 (0.73%) | 120 (0.62%) |
| Education |  |  |  |  |
| below college | 67921 (14.69%) | 28778 (10.29%) | 22805 (12.66%) | 3088 (16.97%) |
| College | 306760 (66.34%) | 194949 (69.72%) | 136724 (75.92%) | 11703 (64.31%) |
| Above college | 87743 (18.97%) | 55879 (19.98%) | 20563 (11.42%) | 3408 (18.73%) |
| Marital status |  |  |  |  |
| Married | 208748 (44.08%) | 101266 (35.42%) | 62167 (33.81%) | 8206 (43.51%) |
| Widow | 84586 (17.86%) | 23559 (8.24%) | 19596 (10.66%) | 2212 (11.73%) |
| Single | 69574 (14.69%) | 67898 (23.75%) | 41298 (22.46%) | 4214 (22.34%) |
| Divorced | 110643 (23.36%) | 93179 (32.59%) | 60799 (33.07%) | 4227 (22.41%) |
| Leading Causes of Deaths* |  |  |  |  |
| COVID-19 | 5651 (1.18%) | 1418 (0.49%) | 1379 (0.73%) | 223 (1.16%) |
| HCC | 14673 (3.06%) | 4719 (1.62%) | 26308 (13.89%) | 3281 (17.03%) |
| Cirrhosis | 200113 (41.78%) | 168104 (57.78%) | 19627 (10.36%) | 1473 (7.64%) |
| Diseases of heart | **48048 (10.03%)** | 11464 (3.94%) | 13191 (6.96%) | 1058 (5.49%) |
| Extrahepatic Cancer | 37547 (7.84%) | 7534 (2.59%) | 24958 (13.18%) | 3269 (16.96%) |
| Accidents (unintentional injuries) | 11665 (2.44%) | 9373 (3.22%) | 5601 (2.96%) | 356 (1.85%) |
| Chronic lower respiratory diseases | 7930 (1.66%) | 2764 (0.95%) | 5102 (2.69%) | 336 (1.74%) |
| Cerebrovascular diseases | 4968 (1.04%) | 1897 (0.65%) | 2209 (1.17%) | 196 (1.02%) |
| Alzheimer disease | 933 (0.19%) | 119 (0.04%) | 343 (0.18%) | 58 (0.30%) |
| Diabetes mellitus | 12237 (2.55%) | 1372 (0.47%) | 3883 (2.05%) | 335 (1.74%) |
| Nephritis, nephrotic syndrome and nephrosis | 3978 (0.83%) | 672 (0.23%) | 1045 (0.55%) | 119 (0.62%) |
| Influenza and pneumonia | 1718 (0.36%) | 487 (0.17%) | 367 (0.19%) | 33 (0.17%) |
| Intentional self-harm (suicide) | 607 (0.13%) | 394 (0.14%) | 268 (0.14%) | 12 (0.06%) |
| Septicemia | 9256 (1.93%) | 2498 (0.86%) | 2061 (1.09%) | 174 (0.90%) |
| Essential hypertension and hypertensive renal disease | 2055 (0.43%) | 629 (0.22%) | 1601 (0.85%) | 139 (0.72%) |
| Parkinson disease | 343 (0.07%) | 41 (0.01%) | 131 (0.07%) | 27 (0.14%) |
| Pneumonitis due to solids and liquids | 1534 (0.32%) | 717 (0.25%) | 381 (0.20%) | 32 (0.17%) |
| Assault (homicide) | 143 (0.03%) | 40 (0.01%) | 33 (0.02%) | 2 (0.01%) |
| Pregnancy, childbirth and the puerperium | 45 (0.01%) | 58 (0.02%) | 14 (0.01%) | 2 (0.01%) |
| Comorbidities** |  |  |  |  |
| COVID | 6869 (1.43%) | 2179 (0.75%) | 1768 (0.93%) | 265 (1.38%) |
| HBV | 0 (0.00%) | 0 (0.00%) | 4406 (2.33%) | 19270 (100.00%) |
| HCV | 0 (0.00%) | 0 (0.00%) | 189398 (100.00%) | 4406 (22.86%) |
| NAFLD | 479011 (100.00%) | 0 (0.00%) | 0 (0.00%) | 0 (0.00%) |
| ALD | 0 (0.00%) | 290952 (100.00%) | 33100 (17.48%) | 2101 (10.90%) |
| HCC | 18485 (3.86%) | 6472 (2.22%) | 30948 (16.34%) | 3663 (19.01%) |
| Cirrhosis | 377396 (78.79%) | 226585 (77.88%) | 106111 (56.03%) | 9475 (49.17%) |
| Extrahepatic Cancer | 50293 (10.50%) | 12269 (4.22%) | 29732 (15.70%) | 3981 (20.66%) |
| Elixhauser Comorbidities** |  |  |  |  |
| Congestive heart failure | 53337 (11.13%) | 15030 (5.17%) | 13022 (6.88%) | 1175 (6.10%) |
| Cardiac arrhythmias | 25446 (5.31%) | 8854 (3.04%) | 7417 (3.92%) | 829 (4.30%) |
| Valvular disease | 7808 (1.63%) | 1969 (0.68%) | 2905 (1.53%) | 256 (1.33%) |
| Pulmonary circulation Disorders | 8796 (1.84%) | 2373 (0.82%) | 3024 (1.60%) | 328 (1.70%) |
| Peripheral vascular disorders | 8482 (1.77%) | 3236 (1.11%) | 3508 (1.85%) | 346 (1.80%) |
| Hypertension, uncomplicated | 37278 (7.78%) | 19882 (6.83%) | 24676 (13.03%) | 2412 (12.52%) |
| Hypertension, complicated | 20839 (4.35%) | 8786 (3.02%) | 9440 (4.98%) | 811 (4.21%) |
| Paralysis | 545 (0.11%) | 179 (0.06%) | 517 (0.27%) | 52 (0.27%) |
| Other neurological disorders | 14859 (3.10%) | 10344 (3.56%) | 6805 (3.59%) | 674 (3.50%) |
| Chronic pulmonary disease | 35245 (7.36%) | 18047 (6.20%) | 23447 (12.38%) | 1607 (8.34%) |
| Diabetes, uncomplicated | 46503 (9.71%) | 14345 (4.93%) | 19618 (10.36%) | 1927 (10.00%) |
| Diabetes, complicated | 15340 (3.20%) | 2685 (0.92%) | 5055 (2.67%) | 541 (2.81%) |
| Hypothyroidism | 3862 (0.81%) | 1320 (0.45%) | 1418 (0.75%) | 165 (0.86%) |
| Renal failure | 55396 (11.56%) | 20088 (6.90%) | 17009 (8.98%) | 1874 (9.72%) |
| Peptic ulcer disease excluding bleeding | 679 (0.14%) | 670 (0.23%) | 331 (0.17%) | 46 (0.24%) |
| AIDS/H1V | 2419 (0.50%) | 805 (0.28%) | 3217 (1.70%) | 784 (4.07%) |
| Lymphoma | 4174 (0.87%) | 608 (0.21%) | 2131 (1.13%) | 627 (3.25%) |
| Metastatic cancer | 12522 (2.61%) | 2990 (1.03%) | 8598 (4.54%) | 1200 (6.23%) |
| Solid tumor without metastasis | 56368 (11.77%) | 16369 (5.63%) | 54900 (28.99%) | 6461 (33.53%) |
| Rheumatoid arthritis/ collagen vascular diseases | 2629 (0.55%) | 508 (0.17%) | 943 (0.50%) | 156 (0.81%) |
| Coagulopathy | 15145 (3.16%) | 13606 (4.68%) | 6127 (3.23%) | 671 (3.48%) |
| Obesity | 12326 (2.57%) | 4466 (1.53%) | 3137 (1.66%) | 219 (1.14%) |
| Weight loss | 8172 (1.71%) | 7064 (2.43%) | 3664 (1.93%) | 397 (2.06%) |
| Fluid and electrolyte disorders | 12497 (2.61%) | 9844 (3.38%) | 4279 (2.26%) | 491 (2.55%) |
| Blood loss anemia | 500 (0.10%) | 399 (0.14%) | 159 (0.08%) | 16 (0.08%) |
| Deficiency anemia | 485 (0.10%) | 406 (0.14%) | 161 (0.09%) | 21 (0.11%) |
| Alcohol abuse | 0 (0.00%) | 260181 (89.42%) | 37583 (19.84%) | 2297 (11.92%) |
| Drug abuse | 4893 (1.02%) | 4380 (1.51%) | 11474 (6.06%) | 791 (4.10%) |
| Psychoses | 661 (0.14%) | 457 (0.16%) | 1291 (0.68%) | 101 (0.52%) |
| Depression | 1757 (0.37%) | 2608 (0.90%) | 2015 (1.06%) | 166 (0.86%) |
| Elixhauser comorbidity index (ECI), mean ± SD | 1.98 ±1.14 | 2.56 ±1.01 | 2.47 ±1.19 | 2.15 ±1.19 |
| Number of Comorbidity = 0 | 103 (0.02%) | 0 (0.00%) | 11 (0.01%) | 688 (3.57%) |
| Number of Comorbidity = 1 | 210904 (44.03%) | 18767 (6.45%) | 39942 (21.09%) | 5551 (28.81%) |
| Number of Comorbidity = 2 | 142096 (29.66%) | 156650 (53.84%) | 71000 (37.49%) | 6833 (35.46%) |
| Number of Comorbidity ≥ 3 | 125908 (26.28%) | 115535 (39.71%) | 78445 (41.42%) | 6198 (32.16%) |
| Abbreviation: SD, Standard deviation; HCC, Hepatocellular carcinoma. * Defined as an underlying cause of death ** Defined as an underlying or contributory cause of death | | | | |

| **S Table 8. Trends in Age-standardized NAFLD-related Death Rate per 100,000 population by Sex and Race/Ethnicity in the Unites States, 2011-2021** | | | | | | | |
| --- | --- | --- | --- | --- | --- | --- | --- |
|  |  | Counts (Rate per 100,000) | | | 2011-2021 | 2011-2019 | 2019-2021 |
| Sex | Group | 2011 | 2019 | 2021 | AAPC (95%CI) | AAPC (95%CI) | AAPC (95%CI) |
| Both | All Race | 34,465 (13.93) | 47,330 (16.32) | 59,230 (20.16) | 3.8 (3.06 - 4.55) | 1.82 (1.29 - 2.35) | 12.13 (7.76 - 16.68) |
|  | non-Hispanic white | 25,416 (13.69) | 34,718 (16.62) | 42,756 (20.71) | 4.18 (3.42 - 4.95) | 2.19 (1.65 - 2.72) | 12.55 (8.03 - 17.25) |
|  | non-Hispanic black | 3,236 (12.15) | 4,002 (12.66) | 4,910 (15.33) | 2.05 (0.74 - 3.37) | 0.46 (-0.57 - 1.5) | 8.66 (3.99 - 13.53) |
|  | Hispanic | 4,648 (21.21) | 6,647 (21.76) | 8,872 (26.72) | 2.75 (1.93 - 3.57) | 0.76 (0.15 - 1.37) | 11.1 (6.36 - 16.05) |
|  | non-Hispanic AIAN | 476 (30.62) | 745 (37.61) | 1,067 (58.16) | 6.95 (5.65 - 8.26) | 2.81 (1.82 - 3.81) | 25.22 (17.29 - 33.7) |
|  | non-Hispanic Asian | 625 (6.27) | 1,082 (7.27) | 1,319 (8.6) | 2.91 (0.86 - 5) | 1.49 (-0.06 - 3.06) | 8.81 (-2.49 - 21.42) |
| Female | All Race | 13,681 (10.3) | 20,136 (12.9) | 25,745 (16.45) | 4.84 (3.99 - 5.7) | 2.76 (2.14 - 3.38) | 13.6 (8.61 - 18.81) |
|  | non-Hispanic white | 10,091 (10.18) | 14,659 (13.15) | 18,592 (17.06) | 5.3 (4.49 - 6.11) | 3.17 (2.6 - 3.75) | 14.28 (9.52 - 19.24) |
|  | non-Hispanic black | 1,222 (8.38) | 1,631 (9.36) | 1,999 (11.47) | 3.15 (1.33 - 5.01) | 1.33 (-0.12 - 2.81) | 10.76 (4.3 - 17.62) |
|  | Hispanic | 1,835 (16.53) | 2,972 (18.55) | 3,892 (22.79) | 3.52 (2.42 - 4.64) | 1.67 (0.83 - 2.51) | 11.3 (4.98 - 18) |
|  | non-Hispanic AIAN | 232 (28.24) | 376 (35.83) | 541 (56.27) | 6.65 (3.5 - 9.9) | 2.43 (0.04 - 4.87) | 25.38 (6.62 - 47.42) |
|  | non-Hispanic Asian | 285 (5.31) | 467 (5.69) | 600 (7.12) | 3.11 (0.84 - 5.44) | 1.66 (-0.07 - 3.41) | 9.15 (-3.33 - 23.24) |
| Male | All Race | 20,784 (18.08) | 27,194 (20.26) | 33,485 (24.36) | 3.04 (2.28 - 3.82) | 1.19 (0.66 - 1.73) | 10.79 (6.28 - 15.49) |
|  | non-Hispanic white | 15,325 (17.67) | 20,059 (20.54) | 24,164 (24.78) | 3.39 (2.63 - 4.16) | 2.17 (1.32 - 3.02) | 8.42 (6.45 - 10.43) |
|  | non-Hispanic black | 2,014 (16.99) | 2,371 (17) | 2,911 (20.36) | 1.69 (-0.26 - 3.67) | 0.23 (-1.92 - 2.42) | 7.73 (2.62 - 13.1) |
|  | Hispanic | 2,813 (26.17) | 3,675 (25.18) | 4,980 (30.71) | 2.09 (0.89 - 3.3) | 0.11 (-0.76 - 1) | 10.4 (3.49 - 17.77) |
|  | non-Hispanic AIAN | 244 (33.18) | 369 (39.58) | 526 (60.01) | 7.24 (3.44 - 11.19) | 3.2 (0.25 - 6.23) | 25.09 (3.1 - 51.78) |
|  | non-Hispanic Asian | 340 (7.34) | 615 (9.22) | 719 (10.31) | 2.34 (0.68 - 4.04) | 2.34 (0.68 - 4.04) | 2.34 (0.68 - 4.04) |
| Abbreviation: AAPC, weighted average annual percent change; AIAN, American Indian or Alaska Native; CI, confidence interval. Standardized to the 2000 US standard population by 10-year age group. AAPC was a weighted average of annual percentage change with weights equal to the length of the detected time segments from the model selected by Joinpoint regression analysis. | | | | | | | |

| **S Table 9. Trends in Age-standardized ALD-related Death Rate per 100,000 population by Sex and Race/Ethnicity in the Unites States, 2011-2021** | | | | | | | |
| --- | --- | --- | --- | --- | --- | --- | --- |
|  |  | Counts (Rate per 100,000) | | | 2011-2021 | 2011-2019 | 2019-2021 |
| Sex | Group | 2011 | 2019 | 2021 | AAPC (95%CI) | AAPC (95%CI) | AAPC (95%CI) |
| Both | All Race | 19,122 (7.76) | 28,959 (10.74) | 40,671 (14.95) | 7.16 (6.39 - 7.93) | 4.54 (3.97 - 5.11) | 18.3 (13.76 - 23.03) |
|  | non-Hispanic white | 13,785 (7.81) | 20,517 (11.26) | 27,942 (15.65) | 7.55 (6.9 - 8.2) | 5.05 (4.58 - 5.52) | 18.17 (14.31 - 22.16) |
|  | non-Hispanic black | 1,490 (5.31) | 2,201 (6.83) | 3,291 (10.1) | 6.74 (4.9 - 8.6) | 3.16 (1.26 - 5.09) | 22.36 (13.78 - 31.58) |
|  | Hispanic | 2,876 (11.02) | 4,721 (13.25) | 6,787 (17.46) | 5.1 (3.08 - 7.16) | 2.74 (1.17 - 4.34) | 15.08 (3.71 - 27.7) |
|  | non-Hispanic AIAN | 651 (37.03) | 988 (51.94) | 1,821 (105.05) | 10.68 (9.16 - 12.22) | 4.24 (3.06 - 5.43) | 40.65 (30.71 - 51.35) |
|  | non-Hispanic Asian | 247 (2.09) | 420 (2.68) | 526 (3.27) | 6.32 (4.42 - 8.25) | 6.32 (4.42 - 8.25) | 6.32 (4.42 - 8.25) |
| Female | All Race | 5,429 (4.34) | 8,697 (6.43) | 12,470 (9.18) | 8.32 (7.08 - 9.58) | 5.64 (4.69 - 6.59) | 19.77 (12.52 - 27.48) |
|  | non-Hispanic white | 4,088 (4.68) | 6,500 (7.32) | 8,980 (10.32) | 8.79 (7.82 - 9.77) | 6.37 (5.64 - 7.1) | 19.05 (13.36 - 25.02) |
|  | non-Hispanic black | 496 (3.24) | 755 (4.4) | 1,170 (6.92) | 8.87 (5.97 - 11.85) | 4.8 (2.56 - 7.09) | 26.79 (9.72 - 46.51) |
|  | Hispanic | 498 (3.56) | 879 (4.76) | 1,336 (6.76) | 6.34 (2.99 - 9.79) | 3.46 (0.8 - 6.19) | 18.68 (0.11 - 40.7) |
|  | non-Hispanic AIAN | 281 (30.75) | 453 (46.25) | 776 (87.96) | 11.27 (9.37 - 13.2) | 4.88 (3.39 - 6.4) | 40.92 (28.67 - 54.33) |
|  | non-Hispanic Asian | 49 (0.77) | 89 (1.05) | 110 (1.29) | 8 (4.94 - 11.16) | 8 (4.94 - 11.16) | 8 (4.94 - 11.16) |
| Male | All Race | 13,693 (11.54) | 20,262 (15.47) | 28,201 (21.11) | 6.54 (5.92 - 7.17) | 4.06 (3.6 - 4.52) | 17.08 (13.39 - 20.89) |
|  | non-Hispanic white | 9,697 (11.2) | 14,017 (15.49) | 18,962 (21.22) | 6.87 (6.25 - 7.51) | 4.42 (3.97 - 4.88) | 17.27 (13.52 - 21.14) |
|  | non-Hispanic black | 994 (7.94) | 1,446 (9.96) | 2,121 (14.02) | 5.85 (4.33 - 7.4) | 2.71 (1.19 - 4.26) | 19.4 (11.6 - 27.74) |
|  | Hispanic | 2,378 (19.5) | 3,842 (22.57) | 5,451 (28.72) | 4.48 (2.53 - 6.47) | 2.43 (0.9 - 3.98) | 13.1 (2.21 - 25.14) |
|  | non-Hispanic AIAN | 370 (44.23) | 535 (58.4) | 1,045 (123.2) | 10.11 (7.82 - 12.45) | 3.7 (1.93 - 5.49) | 39.99 (25.15 - 56.59) |
|  | non-Hispanic Asian | 198 (3.68) | 332 (4.65) | 417 (5.58) | 5.76 (3.77 - 7.78) | 5.76 (3.77 - 7.78) | 5.76 (3.77 - 7.78) |
| Abbreviation: AAPC, weighted average annual percent change; AIAN, American Indian or Alaska Native; CI, confidence interval. Standardized to the 2000 US standard population by 10-year age group. AAPC was a weighted average of annual percentage change with weights equal to the length of the detected time segments from the model selected by Joinpoint regression analysis. | | | | | | | |

| **S Table 10. Trends in Age-standardized HCV-related Death Rate per 100,000 population by Sex and Race/Ethnicity in the Unites States, 2011-2021** | | | | | | | |
| --- | --- | --- | --- | --- | --- | --- | --- |
|  |  | Counts (Rate per 100,000) | | | 2011-2021 | 2011-2019 | 2019-2021 |
| Sex | Group | 2011 | 2019 | 2021 | AAPC (95%CI) | AAPC (95%CI) | AAPC (95%CI) |
| Both | All Race | 17,717 (6.83) | 14,313 (4.72) | 13,950 (4.49) | -4.11 (-5.4 - -2.8) | -4.48 (-5.66 - -3.29) | -2.6 (-9.66 - 5) |
|  | non-Hispanic white | 11,196 (5.94) | 9,101 (4.37) | 8,793 (4.22) | -3.38 (-4.44 - -2.31) | -3.86 (-4.82 - -2.89) | -1.43 (-7.31 - 4.82) |
|  | non-Hispanic black | 3,166 (11.15) | 2,653 (7.7) | 2,446 (7.06) | -4.65 (-6.19 - -3.08) | -3.83 (-5.52 - -2.1) | -7.86 (-9.88 - -5.79) |
|  | Hispanic | 2,603 (10.3) | 1,870 (5.47) | 2,004 (5.45) | -6.23 (-7.65 - -4.79) | -7.32 (-8.6 - -6.01) | -1.77 (-9.72 - 6.89) |
|  | non-Hispanic AIAN | 240 (13.34) | 260 (12.15) | 281 (14.05) | -0.85 (-2.68 - 1.02) | -0.85 (-2.68 - 1.02) | -0.85 (-2.68 - 1.02) |
|  | non-Hispanic Asian | 441 (4.3) | 320 (2.1) | 293 (1.83) | -9.18 (-10.5 - -7.83) | -9.18 (-10.5 - -7.83) | -9.18 (-10.5 - -7.83) |
| Female | All Race | 5,068 (3.79) | 4,030 (2.61) | 4,105 (2.61) | -3.74 (-5.25 - -2.2) | -4.47 (-5.85 - -3.07) | -0.73 (-9.06 - 8.36) |
|  | non-Hispanic white | 3,138 (3.27) | 2,576 (2.5) | 2,644 (2.6) | -2.43 (-3.75 - -1.09) | -3.22 (-4.42 - -2) | 0.81 (-6.51 - 8.7) |
|  | non-Hispanic black | 932 (6.09) | 728 (3.87) | 705 (3.68) | -4.94 (-7.82 - -1.98) | -5.5 (-8.44 - -2.46) | -2.69 (-15.48 - 12.04) |
|  | Hispanic | 720 (5.78) | 491 (2.81) | 485 (2.6) | -8 (-10.13 - -5.81) | -7.53 (-9.98 - -5.01) | -9.84 (-11.96 - -7.66) |
|  | non-Hispanic AIAN | 82 (8.95) | 97 (8.78) | 108 (10.65) | 0.19 (-2.17 - 2.6) | 0.19 (-2.17 - 2.6) | 0.19 (-2.17 - 2.6) |
|  | non-Hispanic Asian | 182 (3.33) | 124 (1.5) | 117 (1.36) | -8.26 (-11.02 - -5.4) | -9.43 (-12.3 - -6.46) | -3.42 (-12.16 - 6.2) |
| Male | All Race | 12,649 (10.09) | 10,283 (7.05) | 9,845 (6.52) | -4.39 (-5.79 - -2.98) | -3.77 (-5.38 - -2.13) | -6.85 (-8.19 - -5.49) |
|  | non-Hispanic white | 8,058 (8.73) | 6,525 (6.35) | 6,149 (5.92) | -3.99 (-5.35 - -2.61) | -3.42 (-4.99 - -1.82) | -6.23 (-7.56 - -4.88) |
|  | non-Hispanic black | 2,234 (17.43) | 1,926 (12.66) | 1,742 (11.47) | -4.17 (-5.95 - -2.35) | -3.34 (-5.3 - -1.34) | -7.39 (-9.73 - -5) |
|  | Hispanic | 1,883 (15.14) | 1,380 (8.4) | 1,519 (8.58) | -5.65 (-6.74 - -4.55) | -6.82 (-7.8 - -5.83) | -0.84 (-7 - 5.73) |
|  | non-Hispanic AIAN | 159 (18.28) | 163 (16.04) | 174 (17.99) | -1.49 (-4.03 - 1.12) | -1.49 (-4.03 - 1.12) | -1.49 (-4.03 - 1.12) |
|  | non-Hispanic Asian | 260 (5.47) | 197 (2.83) | 177 (2.4) | -8.33 (-9.33 - -7.33) | -8.33 (-9.33 - -7.33) | -8.33 (-9.33 - -7.33) |
| Abbreviation: AAPC, weighted average annual percent change; AIAN, American Indian or Alaska Native; CI, confidence interval. Standardized to the 2000 US standard population by 10-year age group. AAPC was a weighted average of annual percentage change with weights equal to the length of the detected time segments from the model selected by Joinpoint regression analysis. | | | | | | | |

| **S Table 11. Trends in Age-standardized HBV-related Death Rate per 100,000 population by Sex and Race/Ethnicity in the Unites States, 2011-2021** | | | | | | | |
| --- | --- | --- | --- | --- | --- | --- | --- |
|  |  | Counts (Rate per 100,000) | | | 2011-2021 | 2011-2019 | 2019-2021 |
| Sex | Group | 2011 | 2019 | 2021 | AAPC (95%CI) | AAPC (95%CI) | AAPC (95%CI) |
| Both | All Race | 1,803 (0.72) | 1,671 (0.59) | 1,756 (0.61) | -1.73 (-3.36 - -0.07) | -2.68 (-3.69 - -1.66) | 2.15 (-7.19 - 12.44) |
|  | non-Hispanic white | 831 (0.45) | 764 (0.39) | 706 (0.37) | _-1.98 (-2.45 - -1.51) | _-1.98 (-2.45 - -1.51) | _-1.98 (-2.45 - -1.51) |
|  | non-Hispanic black | 373 (1.38) | 293 (0.92) | 310 (0.96) | -3.73 (-5.08 - -2.36) | -3.73 (-5.08 - -2.36) | -3.73 (-5.08 - -2.36) |
|  | Hispanic | 162 (0.67) | 118 (0.37) | 146 (0.41) | -4.47 (-8.95 - 0.23) | -6.19 (-11.39 - -0.67) | 2.71 (-4.22 - 10.14) |
|  | non-Hispanic AIAN | 10 (0.62) | 21 (1.03) | 17 (0.82) | 2.3 (-2.59 - 7.42) | 2.3 (-2.59 - 7.42) | 2.3 (-2.59 - 7.42) |
|  | non-Hispanic Asian | 422 (3.78) | 467 (3) | 565 (3.56) | -0.92 (-4.12 - 2.4) | -3.23 (-5.39 - -1.02) | 8.91 (-9.42 - 30.94) |
| Female | All Race | 483 (0.37) | 416 (0.28) | 489 (0.33) | -1.57 (-4.89 - 1.86) | -3.41 (-5.45 - -1.31) | 6.12 (-12.76 - 29.08) |
|  | non-Hispanic white | 224 (0.23) | 183 (0.19) | 201 (0.21) | -1.65 (-3 - -0.28) | -1.65 (-3 - -0.28) | -1.65 (-3 - -0.28) |
|  | non-Hispanic black | 95 (0.65) | 71 (0.42) | 67 (0.38) | -5.49 (-7.36 - -3.6) | -5.49 (-7.36 - -3.6) | -5.49 (-7.36 - -3.6) |
|  | Hispanic | 41 (0.32) | 33 (0.19) | 45 (0.26) | -4.63 (-9.2 - 0.18) | -4.63 (-9.2 - 0.18) | -4.63 (-9.2 - 0.18) |
|  | non-Hispanic AIAN | 6 (0.7) | 8 (0.78) | 8 (0.73) | 1.01 (-5.45 - 7.92) | 1.01 (-5.45 - 7.92) | 1.01 (-5.45 - 7.92) |
|  | non-Hispanic Asian | 118 (2.02) | 123 (1.42) | 165 (1.92) | -0.77 (-4.6 - 3.21) | -4.05 (-6.65 - -1.38) | 13.52 (-8.88 - 41.41) |
| Male | All Race | 1,320 (1.12) | 1,255 (0.93) | 1,267 (0.92) | -1.99 (-2.59 - -1.39) | -1.99 (-2.59 - -1.39) | -1.99 (-2.59 - -1.39) |
|  | non-Hispanic white | 608 (0.68) | 581 (0.62) | 506 (0.53) | -2.43 (-3.93 - -0.9) | -1.64 (-2.53 - -0.73) | -5.52 (-13.62 - 3.34) |
|  | non-Hispanic black | 279 (2.3) | 222 (1.53) | 243 (1.66) | -3.09 (-4.75 - -1.4) | -3.09 (-4.75 - -1.4) | -3.09 (-4.75 - -1.4) |
|  | Hispanic | 122 (1.08) | 86 (0.58) | 102 (0.58) | -5.7 (-8 - -3.35) | -5.7 (-8 - -3.35) | -5.7 (-8 - -3.35) |
|  | non-Hispanic AIAN | 7 (0.91) | 15 (1.52) | 12 (1.25) | 1.04 (-3.15 - 5.41) | 1.04 (-3.15 - 5.41) | 1.04 (-3.15 - 5.41) |
|  | non-Hispanic Asian | 304 (5.9) | 345 (4.98) | 401 (5.54) | -0.96 (-3.97 - 2.14) | -2.76 (-4.79 - -0.69) | 6.57 (-10.35 - 26.69) |
| Abbreviation: AAPC, weighted average annual percent change; AIAN, American Indian or Alaska Native; CI, confidence interval. Standardized to the 2000 US standard population by 10-year age group. AAPC was a weighted average of annual percentage change with weights equal to the length of the detected time segments from the model selected by Joinpoint regression analysis. | | | | | | | |
